# Importance and spatial patterns of invisible fisheries in Amazonian clear‐water rivers as revealed by fisher knowledge and collaboration

**DOI:** 10.1111/cobi.70164

**Published:** 2025-10-26

**Authors:** Renato A. M. Silvano, Kaluan C. Vieira, Paula E. R. Pereyra, Luís H. Tomazoni‐Silva, Ivan A. Alves, Jaqueline G. Bezerra, Márcia C. F. Dutra, Friedrich W. Keppeler, Carolina B. Nunes, Cristiane V. Cunha, Gustavo Hallwass

**Affiliations:** ^1^ Programa de Pós‐Graduação em Ecologia e Departamento de Ecologia Universidade Federal do Rio Grande do Sul (UFRGS) Porto Alegre Brazil; ^2^ Fisheries and Food Institute (FIFO) Rio de Janeiro Brazil; ^3^ Programa de Pós‐Graduação em Ecologia Aquática e Pesca Universidade Federal do Pará (UFPA) Belém Brazil; ^4^ Center for Limnology University of Wisconsin–Madison Madison Wisconsin USA; ^5^ Núcleo de Ecologia Aquática e Pesca da Amazônia Universidade Federal do Pará (UFPA) Belém Brazil; ^6^ Faculdade de Educação do Campo Universidade Federal do Sul e Sudeste do Pará (UNIFESSPA) Marabá Brazil; ^7^ Instituto de Ciência, Tecnologia e Inovação, Programa de Pós‐Graduação em Ecologia Aplicada Universidade Federal de Lavras (UFLA) Lavras Brazil

**Keywords:** Amazon Basin, food security, hydropower, local ecological knowledge, participatory monitoring, protected areas, small‐scale fisheries, sustainability, traditional knowledge, áreas protegidas, conocimiento ecológico local, conocimiento tradicional, cuenca amazónica, energía hidroeléctrica, monitoreo participativo, pesca a pequeña escala, seguridad alimenticia, sustentabilidad, 粮食安全, 可持续性, 参与式监测, 水电, 传统知识, 保护地, 亚马逊流域, 小型渔业, 当地生态知识

## Abstract

The Brazilian Amazon contains the world's most diverse fish assemblages. These assemblages can be affected by freshwater fisheries, which provide food and income for riverine people, and by accelerating environmental change. We collaborated with local fishers to provide a comprehensive assessment of the spatial patterns of fish use in 3 clear‐water rivers in the Brazilian Amazon: the Tapajos, Trombetas, and Tocantins. We interviewed 638 fishers in 39 communities about fish use for domestic consumption or sale, daily catches per fisher, and catch per unit effort (CPUE). We then assessed the influence of river identity, protected areas (PAs), forest cover, and landscape complexity (independent variables) on catches and CPUE estimated from interviews (response variables) through linear models. We also analyzed data from participatory catch monitoring in 21 communities along the Tapajos River (5668 fish landings). Twenty‐one fish species were the most harvested and cited by interview respondents, 16 of which were migratory fishes, accounting for 82% of catches in the Tapajos River. According to fishers, daily fish catches per fisher were higher outside PAs (effect size 0.33) than inside, whereas CPUE was higher inside PAs than outside (−0.27). Catches were negatively associated with forest cover (−0.20), whereas river landscape complexity was positively associated with fish catch (0.96) and CPUE (0.66). These results can support management strategies, from regional to large scales, by reinforcing the relevance of PAs in clear‐water rivers and showing the influence of landscape on fish catches. Our collaboration with fishers provided robust baseline data that can be used to inform inclusive, precautionary, and adaptive policies for conservation of threatened rivers.

## Graphical abstract

The graphical abstract highlights the research collaboration with fishers through interviews to record fishers' knowledge and participatory monitoring, to provide data on small‐scale fisheries in three clear water rivers (Trombetas, Tapajos and Tocantins). The main results show the influence of protected areas and landscape structure on fish catches, the relevance of migratory fishes to the studied fisheries, the importance of these fisheries for food provision, and their vulnerability to environmental changes, such as dams.

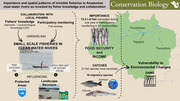

## Abstract (es)

Patrones espaciales e importancia de las pesquerías invisibles en los ríos amazónicos de aguas cristalinas como lo revelaron el conocimiento de los pescadores y la colaboración

**Resumen:**

La Amazonia brasileña alberga las comunidades de peces más diversas del mundo. Estas comunidades pueden verse afectadas por la pesca en aguas dulces, que proporciona alimento e ingresos a las poblaciones ribereñas, y por la aceleración del cambio ambiental. Colaboramos con pescadores locales para realizar una evaluación exhaustiva de los patrones espaciales del uso de los peces en tres ríos de aguas cristalinas de la Amazonia brasileña: el Tapajós, el Trombetas y el Tocantins. Entrevistamos a 638 pescadores de 39 comunidades sobre el uso de los peces para el consumo doméstico o la venta, las capturas diarias por pescador y las capturas por unidad de esfuerzo (CPUE). A continuación, evaluamos la influencia de la identidad del río, las áreas protegidas (AP), la cobertura forestal y la complejidad del paisaje (variables independientes) en las capturas y la CPUE estimadas a partir de las entrevistas (variables de respuesta) mediante modelos lineales. También analizamos los datos del seguimiento participativo de las capturas en 21 comunidades a lo largo del río Tapajós (5668 desembarques de pescado). Las 21 especies de peces más capturadas y citadas por los encuestados eran, en su mayoría, peces migratorios, que representaban el 82% de las capturas en el río Tapajós. Según los pescadores, las capturas diarias por pescador eran mayores fuera de las áreas protegidas (tamaño del efecto 0.33) que dentro de ellas, mientras que la CPUE era mayor dentro de las áreas protegidas que fuera de ellas (‐0.27). Las capturas se asociaron negativamente con la cobertura forestal (‐0,20), mientras que la complejidad del paisaje fluvial se asoció positivamente con las capturas de peces (0.96) y la CPUE (0.66). Estos resultados pueden respaldar las estrategias de gestión, desde la escala regional hasta la gran escala, al reforzar la relevancia de las áreas protegidas en los ríos de aguas cristalinas y mostrar la influencia del paisaje en las capturas de peces. Nuestra colaboración con los pescadores proporcionó datos de referencia sólidos que pueden utilizarse para informar políticas inclusivas, precautorias y adaptativas para la conservación de los ríos amenazados.

## 通过渔民的知识与合作揭示亚马逊清水河隐形渔业的重要性及空间格局

**【摘要】** 巴西亚马逊拥有世界上多样性最高的鱼类群落。淡水渔业为沿岸社区提供了食物和收入, 但淡水渔业和快速的环境变化也会影响鱼类群落的续存。我们与巴西亚马逊地区渔民合作, 对当地三条清水河(塔帕若斯河、特隆贝塔斯河和托坎廷斯河)鱼类资源利用的空间格局进行了全面评估。我们对39个社区的638名渔民进行了访谈, 内容涉及用于家庭消费或销售的鱼类资源利用情况、渔民的人均日渔获量, 以及单位工作量的渔获量(catch per unit effort, CPUE)。随后, 研究通过线性模型评估了河流特征、保护地、森林覆盖度和景观复杂度(自变量)对通过访谈估算的渔获量及CPUE(响应变量)的影响。我们还分析了塔帕若斯河沿岸21个社区的参与式渔获监测数据(5,668条渔获)。受访者提到捕捞最多的鱼类有21种, 其中16种为洄游鱼类, 占塔帕若斯河渔获量的82%。据渔民称, 在保护地外的渔民人均日渔获量高于保护地内(效应量为0.33), 而在保护地内的CPUE则高于保护地外(−0.27)。渔获量还与森林覆盖度呈负相关(−0.20), 而河流景观复杂度则与渔获量(0.96)和CPUE(0.66)呈正相关。这些结果强调了保护地对清水河的重要性, 并展示了景观对渔获量的影响, 从而有助于支持从区域到大尺度的管理策略。我们与渔民的合作提供了稳健的基线数据, 可以为旨在保护受威胁河流的包容性、预防性和适应性政策提供信息。**【翻译:胡怡思;审校:聂永刚】**

## INTRODUCTION

Freshwater, small‐scale fisheries (SSFs) contribute to food security, livelihoods, national economies, and poverty alleviation worldwide (Bartley et al., 2015; Funge‐Smith & Bennett, 2019; Lynch et al., 2020). However, assessing the sustainability of freshwater SSFs is challenging due to limited monitoring and the high variability of fishing practices and socioecological contexts (IPBES, 2022). In tropical regions, fishers rely on a diverse range of fish species (Heilpern et al., 2022), and effective comanagement with fishing communities is critical to achieve sustainability and conservation goals (Campos‐Silva et al., 2018). Moreover, freshwater fishes are among the most imperiled vertebrates globally (Albert et al., 2021), and freshwater ecosystems are increasingly subject to cumulative environmental impacts (Reid et al., 2019) that threaten fish diversity and the sustainability of freshwater SSFs.

The Brazilian Amazon harbors the richest freshwater fish diversity in the world (Dagosta & De Pinna, 2019) and encompasses the largest river basin globally (6,869,000 km^2^) (Goulding et al., 2003). Freshwater SSFs provide essential food and income throughout the Brazilian Amazon (Hallwass & Silvano, 2016), where riverine people have high fish consumption rates (Isaac et al., 2015); yet, ecological information on harvested fish species remains scarce (Begossi et al., 2019). Many of the most important fish species for Amazonian fisheries are migratory, meaning a large portion of their populations undertake periodic or seasonal movements between 2 or more distinct habitats (Herrera‐R. et al., 2024). These harvested migratory fish are particularly challenging to manage (Duponchelle et al., 2021; Hallwass & Silvano, 2016) and are especially vulnerable to the effects of dams (Hallwass et al., 2013; Van Damme et al., 2019).

The Amazon basin is being subjected to increasing anthropogenic pressures (Albert et al., 2023), including deforestation, river damming for hydropower, overfishing, and mercury poisoning of fish and people, all of which can negatively affect Amazonian fish populations and fisheries (Castello et al., 2013; de Vasconcellos et al., 2021; Santos et al., 2020; Winemiller et al., 2016). Conversely, fishing yields can be positively influenced by forest cover (Castello et al., 2018), although the effects of other landscape variables, such as landscape complexity, have been less extensively studied (Keppeler et al., 2018).

Clear‐water rivers account for approximately 27% of the Brazilian Amazon area (Goulding et al., 2003) and differ from one another and from white‐ and black‐water Amazonian rivers in terms of environmental conditions and extent of research. Fisheries in clear‐water rivers often exhibit lower fish yields because these systems are typically less productive (oligotrophic) compared with white‐water rivers, such as the lower Amazon or Madeira (Arantes et al., 2018; Castello et al., 2018, 2013; Hallwass et al., 2023). Additionally, clear‐water rivers are considered the most threatened aquatic ecosystems in the Brazilian Amazon (Latrubesse et al., 2017). They are at the forefront of infrastructure development, particularly dam construction, due to favorable conditions for hydropower generation, including rapids and low sediment loads (Flecker et al., 2022). Despite this, clear‐water rivers have received less attention in conservation initiatives and fisheries management policies than more productive white‐water rivers. This oversight may have led to SSFs and fish diversity in Amazon tributaries being neglected in dam development planning and implementation (Athayde et al., 2019; Doria et al., 2017; Runde et al., 2020). Consequently, information on fish use by local people, whether for commercial sale or domestic consumption, is urgently needed to support fish conservation, poverty alleviation, and assessment of environmental change impacts, particularly in understudied and threatened rivers that support high fish diversity.

An inclusive conservation approach, grounded in collaborations between researchers and local communities, can enhance socioeconomic welfare while protecting aquatic ecosystems in the Brazilian Amazon (Lopes et al., 2021). This can be achieved through the establishment of protected areas (PAs) that allow sustainable use and are managed cooperatively with local people or through a fishing agreement, a bottom‐up fisheries management system that emerged in the Brazilian Amazon (Almeida et al., 2009; Campos‐Silva & Peres, 2016; Campos‐Silva et al., 2018, 2021; Franco et al., 2021; Lopes et al., 2011; Silvano et al., 2014). Sustainable‐use PAs, which allow local people to harvest natural resources under comanagement regimes, have been widely implemented in Brazil and across the Amazon (Lopes et al., 2011). Collaborative research approaches, including participatory monitoring and studies of local ecological knowledge (LEK), which encompasses fisher knowledge, can provide much needed data in a timely and cost‐effective manner (Keppeler et al., 2020; Pereyra et al., 2023; Stenekes et al., 2020). Such collaborations can help fill critical research gaps and inform conservation and sustainable management policies (Danielsen et al., 2021; Esmail et al., 2023; Hallwass et al., 2024), particularly for poorly documented SSFs (Silvano et al., 2023). Previous partnerships with fishers, established through interviews to document LEK and participatory fisheries monitoring, have generated valuable information on fish migrations, feeding habits, temporal patterns in fish abundance, and fishing strategies in the Brazilian Amazon (Hallwass et al., 2023; Hallwass, Schiavetti, et al., 2020; Keppeler et al., 2020; Nunes et al., 2019; Pereyra et al., 2023, 2021; Poissant et al., 2024).

We collaborated with fishers, drawing on their knowledge and voluntary monitoring efforts to compile fisheries data and examine the drivers influencing fish catches in 3 Amazonian clear‐water rivers, which are increasingly being threatened by environmental changes. First, we assessed the composition of the most used fish species for domestic consumption and commercial sale and evaluated the influence of river and PA status on this composition. Second, using interview and participatory monitoring data, we analyzed the harvest of migratory fish species within the broader composition of harvested fishes, given the ecological importance and vulnerability of migratory fishes. Third, we investigated the influence of river, PA, age of fisher, and landscape variables on the quantity (weight) of fish caught and catch per unit effort (CPUE) based on fishers’ LEK collected in interviews.

## METHODS

### Study area

**FIGURE 1:**
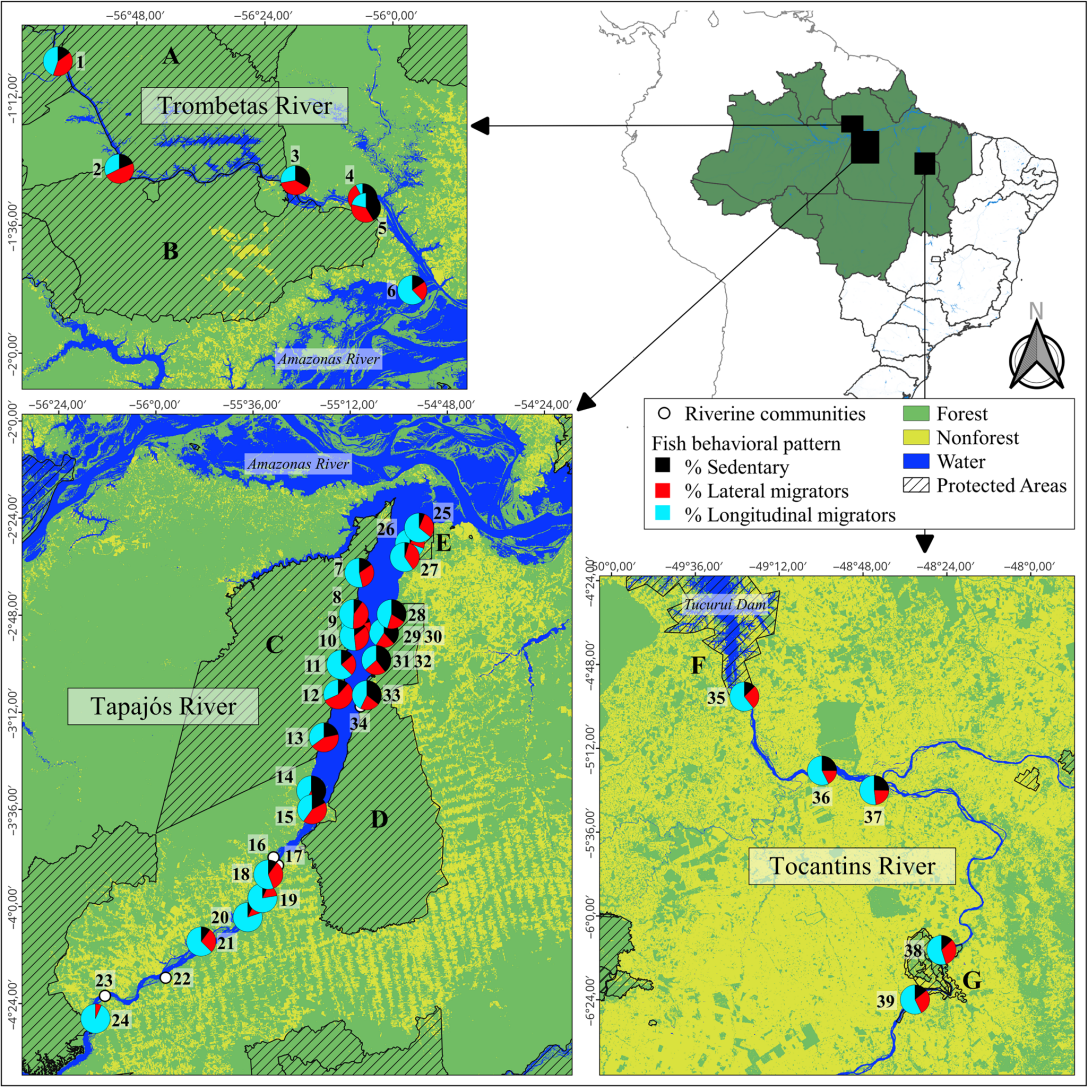
Location of the 3 studied rivers, protected areas (capital letters), and fishing communities (numbers) in the Brazilian Amazon, protected area boundaries, major land‐cover categories, and percentage of fish catches or citations per community categorized by fish behavioral patterns (details in Methods) (dots, communities without fish landing data in the Tapajos). Names of numbered communities, protected areas, and the quantity of interviews and fish landings are in Table 1. Migratory fish percentages are based on fish landings (Tapajos) or fisher interviews (Trombetas and Tocantins).

We conducted our study in the Brazilian Amazon along the Tapajos, Trombetas, and Tocantins Rivers. Thirty‐nine riverine communities were included: 6 in the Trombetas, 28 in the Tapajos, and 5 in the Tocantins–Araguaia (Figure 1). Although some communities were located on the Araguaia River, near its confluence with the Tocantins (Figure 1), we refer to the Tocantins–Araguaia River basin as the Tocantins River. These 3 large rivers, characterized by low concentrations of nutrients and suspended sediments, are classified as clear‐water or oligotrophic rivers and are tributaries of the larger Amazon River (Junk et al., 2011). The relatively less studied Trombetas River contains extensive PAs and preserved forests (Nunes et al., 2023). The Tapajos River features 2 large sustainable‐use PAs and preserved forests in its lower reaches; its middle stretch has been subjected to deforestation and mercury contamination from gold mining (Capitani et al., 2021; de Vasconcellos et al., 2021; Keppeler et al., 2018, 2017; Nunes et al., 2023). In contrast, the Tocantins River has undergone drastic land‐cover changes, losing 43% of its forested area from 1985 to 2019 (Nunes et al., 2023), and has been affected by the construction of 7 dams (Hallwass et al., 2013; Pelicice et al., 2021; Swanson & Bohlman, 2021). Across these rivers, fishers primarily use small boats and gillnets. Catches are destined either for domestic consumption (subsistence) or for sale in local and regional markets (Hallwass et al., 2023; Hallwass, Silva, et al., 2020; Isaac et al., 2015; Runde et al., 2020).

Of the 39 communities we surveyed, 22 were in sustainable‐use PAs (Figure 1; Table 1): the Trombetas River Biological Reserve and Saracá‐Taquera National Forest in the Trombetas River (A and B in Figure 1); the Tapajos‐Arapiuns Extractive Reserve, Tapajos National Forest, and Alter do Chão Environmental Protection Area (EPA) in the Tapajos River (C, D, and E in Figure 1); and the Lago de Tucuruí and São Geraldo do Araguaia EPAs in the Tocantins River (F and G in Figure 1). These PAs differed in age and in the regulations governing fisheries. The EPAs were typically less restrictive than other PA categories (Table 1).

**TABLE 1:** Relative to protected areas (PAs) (inside or outside), the location of studied fishing communities along 3 rivers; the year of PA establishment and fisheries regulations for each PA; number of individual fisher interviews providing data on catch, catch per unit effort, and fish use; and the number of recorded fishing trips (or fish landings) from participatory monitoring.

					Interviews			Fish landings		
River and PA (year)^a^	Protected area code^b^ and fishing regulations^c^	Community number^d^	Location relative to PA	Community	Catch (kg)	Catch per unit effort^e^	Fish use^f^	*n* ^g^	Catch (kg)	kg/landing
Trombetas										
Trombetas River Biological Reserve (1979)^h^	A Only residents from PA can fish Subsistence fishing only Transportation of fish prohibited Damaging gear prohibited (but not defined)	1	Inside	Cachoeira Porteira^i^	16	16	15			
Saracá‐Taquera National Forest (1989)	B Damaging gear prohibited (but not defined), no other rules	2	Inside	Tapagem	8	7	8			
		3	Outside	Mussura	4	3	5			
		4	Outside	Varjão^i^	13	13	16			
		5	Outside	São Benedito Carimum	16	16	16			
		6	Outside	Aimim and Santa Maria Goretti	7	7	7			
Tapajos										
Tapajós‐Arapiuns Extractive Reserve (1998)	C Only residents from PA can fish Traditional gears only (arrow, hook and line, cast net, spear, fixed gillnet) Beating, fish poison, explosives, dragnets, blocking the entrance of streams and lakes, diving fishing prohibited Gillnets and cast nets prohibited in lakes during dry season in some communities	7	Inside	Capichauã^i^	21	21	19	165	978.9	5.9
		8	Inside	Vila do Amorim			20	121	1098.7	9.1
		9	Inside	Parauá^i^	31	31	24	365	7889.0	21.6
		10	Inside	Surucuá^j^	16	16	24	259	1567.3	6.1
		11	Inside	Jauarituba^j^	25	25	20	440	3323.2	7.6
		12	Inside	Boim^i^	11	11	23	199	891.6	4.5
		13	Inside	Cametá^j^	32	32	25	396	3003.3	7.6
		14	Outside	Apacê^i^	24	24	20	363	4951	13.6
		15	Outside	Santa Cruz and Daniel de Carvalho^i^	32	32	25	173	720	4.2
		16	Outside	Uricurituba	9	8	9			
		17	Outside	Fordlândia^i^	6	6	7			
		18	Outside	Cauaçuepá^i^	25	25	12	123	1687.8	13.7
		19	Outside	Brasília Legal^i^	37	37	19	357	10,715.3	30.0
		20	Outside	Barreiras^i^	35	35	23	567	16,757.1	29.6
		21	Outside	Pedra Branca^i^	28	27	16	154	2257	14.7
		22	Outside	Miritituba and Itaituba^i^	23	23	19			
		23	Outside	Boa Vista do Tapajós and Canaã			10			
		24	Outside	São Luiz do Tapajós^i^	17	16	13	190	2853.3	15.0
Alter do Chão Environmental Protection Area (2003)	D Beating water to drive fish to nets, fish poison, explosives, dragnets, diving for fish prohibited Gillnets limited to a maximum length of 150 m, minimum mesh size 35 mm except during high water season, when minimum mesh size is 25 mm	25	Inside	Ponta de Pedras^j^	16	16	14	456	7260.2	15.9
		26	Inside	Alter do Chão^j^	18	18	20	392	3382.9	8.6
		27	Inside	Pindobal^i^	6	6	20	143	483.6	3.38
Tapajós National Forest (1974)	E Only residents from PA can fish Subsistence fishing only Traditional gears only (arrow, hook and line, cast net, spear, fixed gillnet) Diving for fish prohibited	28	Inside	Maguari^i^	13	13	18	68	216.9	3.2
		29	Inside	Acaratinga^i^	7	7	13	161	374.2	2.3
		30	Inside	São Domingos^i^	13	13	12			
		31	Inside	Piquiatuba^i^	13	13	18	209	800.2	3.83
		32	Inside	Pedreira^i^	13	13	10			
		33	Inside	Pini^i^	17	17	8	367	1234.5	3.36
		34	Inside	Prainha^i^	9	9	7			
Tocantins										
Lago de Tucuruí Environmental Protection Area (2002)	F Nets, cast nets, spear and treble hook, fish traps, other fixed gear that blocks fish passage, diving for fish (spearfishing), light and sound devices, buoys and other floating devices, baiting to attract fish, beating water to drive fish to nets, shocking, poisoning, use of explosives prohibited	35	Inside	Vila Tauiry^i^	24	19	25			
		36	Outside	Espírito Santo^i^	13	12	17			
		37	Outside	Apinajés^i^	20	20	18			
São Geraldo do Araguaia Environmental Protection Area (1996)	G No specific fishing rules	38	Inside	Santa Cruz^i^	15	15	11			
		39	Inside	São Geraldo do Araguaia	5	5	4			
Total		39			638	627	610	5668	72,446	10.7^k^

^a^ Information from Ministério do Meio Ambiente; Sistema Nacional de Unidades de Conservação da Natureza (Lei n° 9.985, de 18 de julho de 2000; Decreto n° 4.340, de 22 de agosto de 2002; Decreto n° 5.746, de 5 de abril de 2006); Plano Estratégico Nacional de Áreas Protegidas (Decreto n° 5.758, de 13 de abril de 2006); and Brasília (https://www.gov.br/mma).

^b^ Letter codes are PAs in Figure 1.

^c^ Information extracted from the management plans of each PA.

^d^ Numbers are on Figure 1 and indicate the location of each community.

^e^ Kilograms of fish per hour of fishing × number of fishers.

^f^ Fish used by fishers for domestic consumption or commerce, according to interviews.

^g^ Total number of recorded fish landings in each studied community.

^h^ Although biological reserves in Brazil usually do not allow resident people the direct use of natural resources, an exception was made in this one to accommodate resident traditional communities, their rights, and their resource needs.

^i^ Community revisited or studied in 2 distinct years (research projects). Numbers of communities studied in each year are in Appendix S1.

^j^ Community revisited or studied in 3 distinct years (research projects). Numbers of communities studied in each year are in Appendix S1.

^k^ Average value from all communities.

Participants in our study were the Amazonian riverine people who lived in small communities and identify as *caboclos*. This cultural group comprises descendants of Indigenous peoples and Portuguese colonizers, immigrants from other Brazilian regions, and descendants of enslaved people, known as *quilombolas*. In these riverine communities, people often engage in multiple economic activities, which vary by season (Estevo et al., 2023). Their primary livelihoods include small‐scale agriculture, animal husbandry, fishing, hunting, handicrafts, and gathering (Begossi et al., 1999; McGrath et al., 2008; Medeiros et al., 2021).

### Data collection

We conducted interviews and participatory monitoring from 2013 to 2023 (Appendix S1). We purposively selected communities (sample sites) with 10 or more houses for which fishing was important, communities willing to participate in the research, and communities located inside and outside PAs. We grouped neighboring communities that had fewer than 5 interviewees, resulting in 39 communities for analyses (Table 1). We revisited 31 of these communities: 6 in 3 different years and 25 in 2 different years (Table 1). For revisited communities, we included only new interviews in our analyses to ensure each interview corresponded to a different fisher.

We focused greater sampling effort along the Tapajos River, where we conducted interviews and participatory monitoring of fish landings. This approach reflected our long‐term research commitment in this river since 2013 and growing concerns about emerging environmental pressures, such as dams and mercury contamination (Faial et al., 2015; Hallwass, Schiavetti, et al., 2020; Pereyra et al., 2024; Runde et al., 2020). Upon arriving at each community, we explained the research to local leaders and requested permission to conduct the study. Leaders then introduced us to fishers who met the minimum criteria for inclusion in the survey: fishing regularly (e.g., weekly) for food or income, living in the region for at least 10 years, and being over 18 years old. We selected additional fishers to be interviewed in a nonrandom, purposive manner with snowball sampling (i.e., we asked interviewed fishers to suggest others to interview) (Hallwass, Schiavetti, et al., 2020; Runde et al., 2020).

We approached each fisher individually, explained the study, and asked for consent to participate in an interview based on a standard and structured questionnaire. During the study period, we conducted several different studies, and questionnaires differed depending on each study's objectives, but all questionnaires included questions on socioeconomic characteristics, local economic activities, fish use (harvested for sale or consumption), and fishing practices (Appendix S2). For the work presented here, we analyzed questions that pertained to fish catches (total catch weight per fisher interviewed on an average fishing day), fishing effort (hours spent fishing and number of participants per trip), and the 5 most relevant fish species used by each interviewee for sale or domestic consumption (some interviewees cited more or fewer than 5 species). We then calculated CPUE as the total catch divided by the product of hours spent fishing and number of fishers per trip (2 measures of effort). This simple CPUE metric has been used widely in previous SSFs research based on interviews or monitoring data (Glaser & Diele, 2004; Hallwass, Schiavetti, et al., 2020; Silvano & Hallwass, 2020). We calculated the average daily fish harvest per fisher by averaging the reported catches from all 638 interviewees (Table 1). Each interview typically lasted about 30 min (Silvano & Hallwass, 2020).

We conducted participatory fisheries monitoring in 21 communities along the Tapajos River (Figure 1; Table 1) in 2013–2014, 2016–2017, and 2021–2022. We invited interviewed fishers who met the minimum criteria (at least 5 years of formal education and fishing at least 3 times a week) to voluntarily record their first 5 fish landings each month for 1 year, following established protocols (Hallwass, Silva, et al., 2020; Keppeler et al., 2020; Silvano & Hallwass, 2020). We trained participating fishers and provided materials (wristwatches, scales, tape measures, pencils) and standardized forms (Appendix S3) for data recording. Fishers registered the total weight of fish caught (in kilograms) by common names of fish provided by each fisher, income from sales, fishing grounds, gears used, vessel type, amount of catch allocated for domestic consumption, and fishing effort (time and crew size).

We used previous records of fish species and their common names from the Tapajos River (Silvano et al., 2022) to assign reported fish names to single species or, when necessary, to species groups, typically at the genus level (Appendix S4). Fishers in the Tapajos River consistently recognize and name many fish species, particularly those commonly harvested, by similar names across different communities (Silvano et al., 2022). We collected the monitoring forms from fishers every 3 months and applied a 3‐step data verification process (Nagl et al., 2021). First, we validated the data during collection by reviewing records with the fishers. Next, we cross‐checked fish length and weight values against the literature (Froese & Pauly, 2019) during data entry. Finally, we checked for outliers during analyses. After verification, we excluded 332 fish landings (5.5% of 6000 recorded) from the analyses. We shared results with participants and community leaders through meetings and printed materials, such as books and folders (available in Portuguese at https://linktr.ee/lehpe.ufrgs). We calculated the average daily fish harvest per fisher by calculating the mean catch of recorded fish landings in each community and then calculating an overall average across all communities (Table 1).

We applied the same procedures for interviews and participatory monitoring in all studied communities. We obtained approvals from the Brazilian Ethics Committee for research involving human participants (CAAE 49885415.1.0000.5347, 82355618.0.0000.5347, 61558022.1.0000.5347, 53197321.0.0000.0171).

### Data analyses

We analyzed the species composition of used fish by organizing a matrix with 39 riverine communities as sampling sites (Table 1) and the 18 most cited fish species in interviews as variables (vectors), based on the percentage of citations (3468 total citations) for each species in each community. We labeled and combined the factors (PAs and rivers) before analysis. We excluded 20 rarely cited fish species, each representing <1% of the total citations, from the data matrix. We applied nonmetric multidimensional scaling ordinations (NMDS) to visually compare the composition of used fish among communities; closer points (communities) indicated similar composition of cited species. The ordination plot displayed vectors (fish species) that most influenced the ordination (correlation >0.5). We used the same dataset to compare the composition of cited fish among the 3 studied rivers and between communities inside and outside PAs in the Tapajos through a 2‐way crossed analysis of similarities (ANOSIM), which reports global *R* values from 0 (no difference) to 1 (complete difference). We could not test the effect of PA on fish composition in the Trombetas and Tocantins Rivers because of the limited number of replicates (communities) inside and outside PAs. We used the Bray–Curtis distance and 1000 permutations for randomization in the NMDS and ANOSIM analyses in PRIMER 6 software (Clarke & Gorley, 2006).

We estimated the proportional catches (by weight) of migratory fish in the Tapajos River based on the migratory behavior of each harvested species (or group of species) with data (catches) from participatory monitoring. In the Trombetas and Tocantins Rivers, where no monitoring was conducted (Figure 1; Table 1), we calculated the proportion of migratory fish based on interview citations. After assigning the common fish names provided by fishers (in interviews or fish landings) to fish species or groups, we classified each cited or landed fish into one of 3 migratory behavior categories (Herrera‐R et al., 2024): longitudinal migrators (species move upstream and downstream along the main channel, typically over long distances); lateral migrators (species move between the river, lakes, and floodplains); and sedentary species (species without migratory movements).

We analyzed landscape attributes to account for habitat complexity around the studied communities. We used land‐use and land‐cover maps from Project MapBiomas in Brazil (Souza & Azevedo, 2017) to consider 3 landscape categories: natural formation (mostly forest), anthropic areas (deforested land or exposed soil from agriculture, pasture, or buildings), and water cover (http://www.mapbiomas.org). We established a 10‐km‐diameter area around the geographical center of each riverine community along the river shore and calculated landscape attributes in this area. We calculated the proportion of water cover as the ratio of water‐covered surface to land area, considering the annual average water surface per pixel (30 m^2^). Similarly, we estimated the proportion of forest cover as the ratio of forested land to deforested areas. To measure river width, we drew a straight line from each community, perpendicular to the riverbank, crossing the riverbed to the opposite shore. We used yearly averages based on satellite images produced every 16 days in 2016 (http://www.mapbiomas.org).

To assess landscape complexity, we calculated the landscape shape index (LSI) (Patton, 1975), which reflected the perimeter‐to‐area ratio (accounting for size and shape) of land masses within a 10‐km radius around each community (Appendix S5). Higher LSI values indicated a relatively complex landscape with intricate (irregular) shapes. This metric was applied in previous studies of Amazonian lakes (Keppeler et al., 2018). For these calculations, we classified areas on MapBiomas maps as either water or land and measured the area and perimeter of land within a 10‐km area centered on each community along the river. We performed these spatial analyses with QGIS 3.28.4 Firenza (QGIS Development Team, 2022) and the R package (R Core Team, 2025) terra (Hijmans et al., 2022). Because LSI, water area, and river width were highly correlated, we used principal component analyses (PCAs) to reduce data dimensionality and address multicollinearity. The first PCA axis explained 93% of the data variation and was positively associated with LSI (axis score 3.62) and negatively associated with water area (−3.69) and river width (−3.65). We used this axis as a composite measure of landscape structure (hereafter, landscape structure [PC1]).

We applied linear models to assess the effects of independent variables (river, fishers’ age, PA, forest cover, and landscape structure [PC1]) on dependent variables derived from fishers’ knowledge (interviews): daily catch per fisher (catches) and CPUE. We built one model for each dependent variable, treating PA (inside or outside) and river (the 3 studied rivers) as categorical variables and age, forest cover, and landscape structure (PC1) as continuous variables. We assumed there was a linear relationship with the response variables and did not include polynomial or interaction terms in the models. To meet the assumptions of normality and homoscedasticity, we transformed catches and CPUE to the log scale. We assessed multicollinearity with generalized variance inflation factors, which indicated that our models had acceptable levels (<5). We included fisher age as a covariate in these analyses, recognizing that older fishers may recall larger historical catches (Sáenz–Arroyo et al., 2005).

After building the global model with all independent variables, we created a model selection table based on the Akaike information criterion corrected for small samples (AIC_c_) by evaluating combinations (subsets) of fixed effect variables. We then applied a model‐averaging procedure (Appendix S5) to estimate the relative importance of each independent variable, with values ranging from 0 (no importance) to 1 (highest importance) (Burnham & Anderson, 2002). We calculated robust parameter estimates, including 95% confidence intervals (CIs), for all variables of interest based on 32 candidate models. We used the conditional average method to derive parameter coefficients and considered predictors (independent variables) significant when their 95% CI did not include zero, indicating a clear positive or negative relationship with the response variable (Burnham & Anderson, 2002). We used the global model to visualize these relationships. To assess the goodness of fit of the best models, we calculated marginal (variance explained by the independent variables alone) and conditional (variance explained by independent and random variables) *R* ^2^ values (Nakagawa & Schielzeth, 2013).

We built the linear models and conducted PCAs, model‐averaging procedures, and regression visualizations in R statistical software with the packages stats (R Core Team, 2025), vegan (Oksanen et al., 2013), MuMIn (Barton, 2009), and visreg (Breheny & Burchett, 2017).

## RESULTS

### Patterns of fish use

The number of interviewed fishers differed among variables (Table 1). In total, 638 fishers provided information on fish catches, and 610 fishers contributed 3468 citations of individual fish used for sale or domestic consumption across the 3 rivers (Table 1). In the Tapajos River, 128 fishers recorded 5668 fish landings that altogether contained 72.4 t of fish during 1 year of participatory monitoring in 21 communities. The average daily fish harvest per fisher was 28.3 kg (SD 75.6) based on interviews and 10.7 kg (8.25) based on fish landings (Table 1). Interviewed fishers cited 38 fish species, and participatory monitoring of fish landings documented 55 species. However, 10 fish species emerged as the most cited in interviews and the most frequently harvested: pescada (*Plagioscion squamosissimus*) (Heckel, 1840), aracu (*Leporinus* spp., *Schizodon* spp.), tucunaré (*Cichla* spp.), jaraqui (*Semaprochilodus* spp.), acaratinga (*Geophagus* spp.), pacu (*Myleus* spp., *Myloplus* spp.), filhote (*Brachyplatystoma filamentosum*) (Lichtenstein, 1819), dourada (*Brachyplatystoma rousseauxii*) (Castelnau, 1855), mapará (*Hypophthalmus* spp.), and sarda (*Pellona* spp.), highlighting their socioeconomic importance (Table 2).

**TABLE 2:** Fishes most cited by fishers during interviews inside and outside protected areas (PAs) in the 3 rivers and most caught in fish landings in the Tapajos River.

Trombetas		Tapajos			Tocantins	
Inside PA	Outside PA	Inside PA	Outside PA	Fisher landings	Inside PA	Outside PA
Pacu	Tucunaré	Pescada	Aracu	Pescada	Aracu	Curimata
Tucunaré	Pacu	Tucunaré	Pescada	Jaraqui	Curimata	Tucunaré
Aracu	Acara	Acaratinga	Jaraqui	Tucunaré	Tucunaré	Pacu
Pescada	Aracu	Aracu	Tucunaré	Filhote	Mandi	Aracu
Filhote	Charuto	Jaraqui	Pacu	Mapará	Pacu	Surubim
Charuto	Pescada	Mapará	Filhote	Pacu	Jau	Piranha
Surubim	Tambaqui	Filhote	Dourada	Aracu		Jaraqui
Mapará	Piranha	Dourada	Mapará	Sarda		Jau
Matrinxã		Pacu	Tambaqui	Dourada		Corvina
		Sarda	Surubim	Acaratinga		Acari

*Note*: Fish are listed in order of importance for each river, based on citations or catches. Fish scientific names are provided in the text and Appendix S4.

**FIGURE 2:**
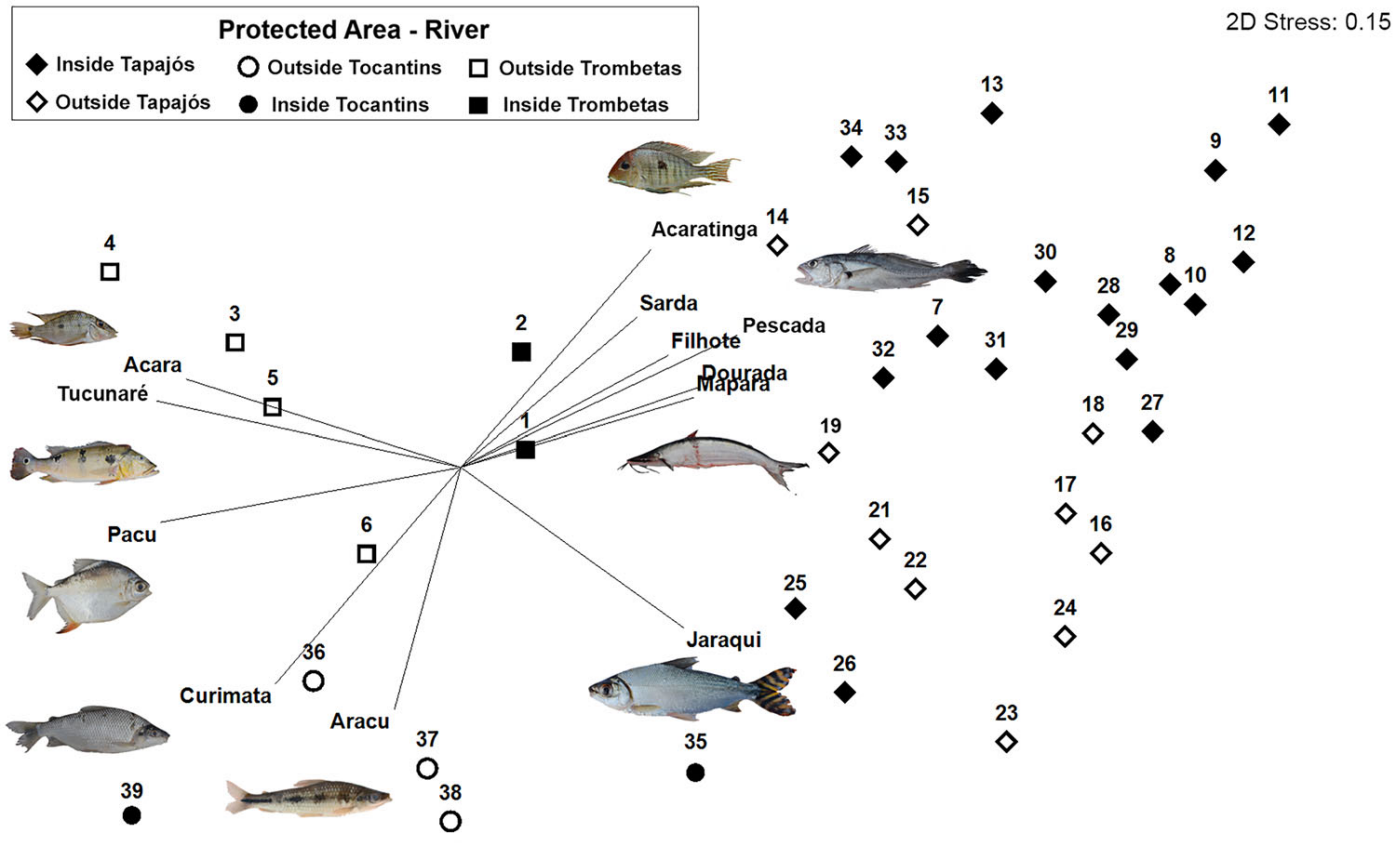
Nonmetric multidimensional scaling ordinations and results of analysis of similarities in which the composition of fish species cited by fishers is compared among the 3 studied rivers and between sites (communities) inside and outside protected areas (points, fishing communities; numbers, community identification numbers in Figure 1 [names in Table 1]; vectors, fish species [scientific names in Appendix S4] most influencing the ordination; correlation values, >0.5 with at least one axis).

The most cited fish in interviews for domestic consumption or sale varied among the 3 rivers (global *R* = 0.87, *p* = 0.001); all pairwise comparisons were significant at *p* < 0.05 (Appendix S6; Figure 2). Although some species, such as tucunaré and aracu, remained consistently important across rivers, the most cited fish were pacu in the Trombetas, pescada in the Tapajos, and curimata (*Prochilodus nigricans*) (Agassiz, 1829) in the Tocantins (Table 2). We also detected differences in fish use among communities inside and outside PAs in the Tapajos River (global *R* = 0.32, *p* = 0.001), suggesting that PAs may influence the fish species used for domestic consumption or sale.

### Migratory fish and fisheries

In most communities, the majority of catches recorded through participatory monitoring in the Tapajos River consisted of migratory fish performing longitudinal or lateral migrations (mean [SD] = 82.3% [13.2]). In communities in the middle Tapajos (community numbers 18–24 in Figure 1), fish catches were predominantly longitudinal migrators. For example, in Barreiras (number 20 in Figure 1b) and Brasília Legal (number 19), migratory fish accounted for 93% of catches, and longitudinal migrators represented 80% and 77%, respectively. In São Luiz do Tapajos (number 24), the most upstream community, all recorded fish catches were migratory species, and 93% were longitudinal migrators. These communities also reported higher fish catches in fish landings (Table 1) and interviews (Appendix S7) than were reported for other communities in the Tapajos River.

Interviews underscored the importance of migratory fish to fisheries in the Trombetas and Tocantins Rivers (Figure 1). On average, migratory fish species accounted for 70.7% (SD 15.2) of cited fish in the Trombetas and 82% (6.6) in the Tocantins. Lateral migrators were among the most cited in the Trombetas, whereas longitudinal migrators dominated the cited species in the Tocantins River (Figure 1).

### Influence of PAs and landscape on fisheries indicators

**FIGURE 3:**
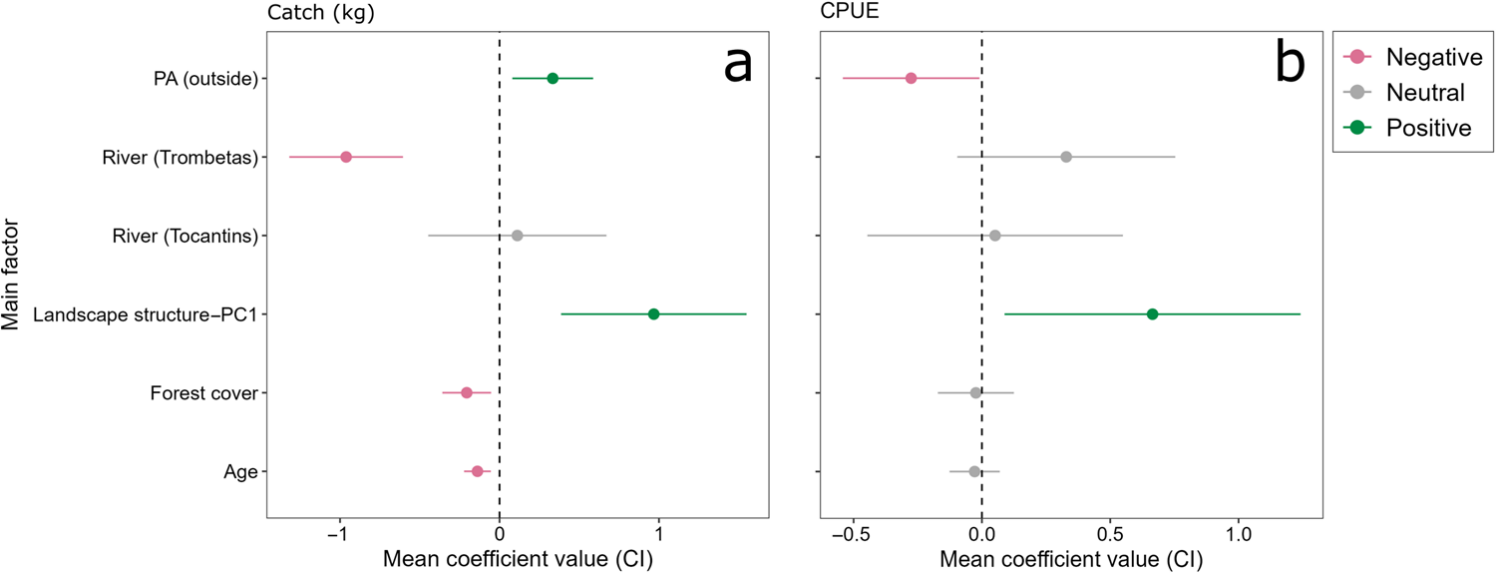
Coefficient slopes and 95% confidence intervals (CIs) from averaged linear models for (a) fish catches and (b) catch per unit effort (CPUE) measured as kilograms of fish caught per hour of fishing (PA, protected area; landscape structure—PC1, first principal component axis of a multivariate analysis combining landscape shape index, water area, and river width; age, interviewee's age; coefficients for categorical variables protected area and river, differences relative to fixed baseline levels inside protected areas and Tapajos River; coefficient intervals not overlapping zero, significant positive [above zero] or negative [below zero] effects).

**FIGURE 4:**
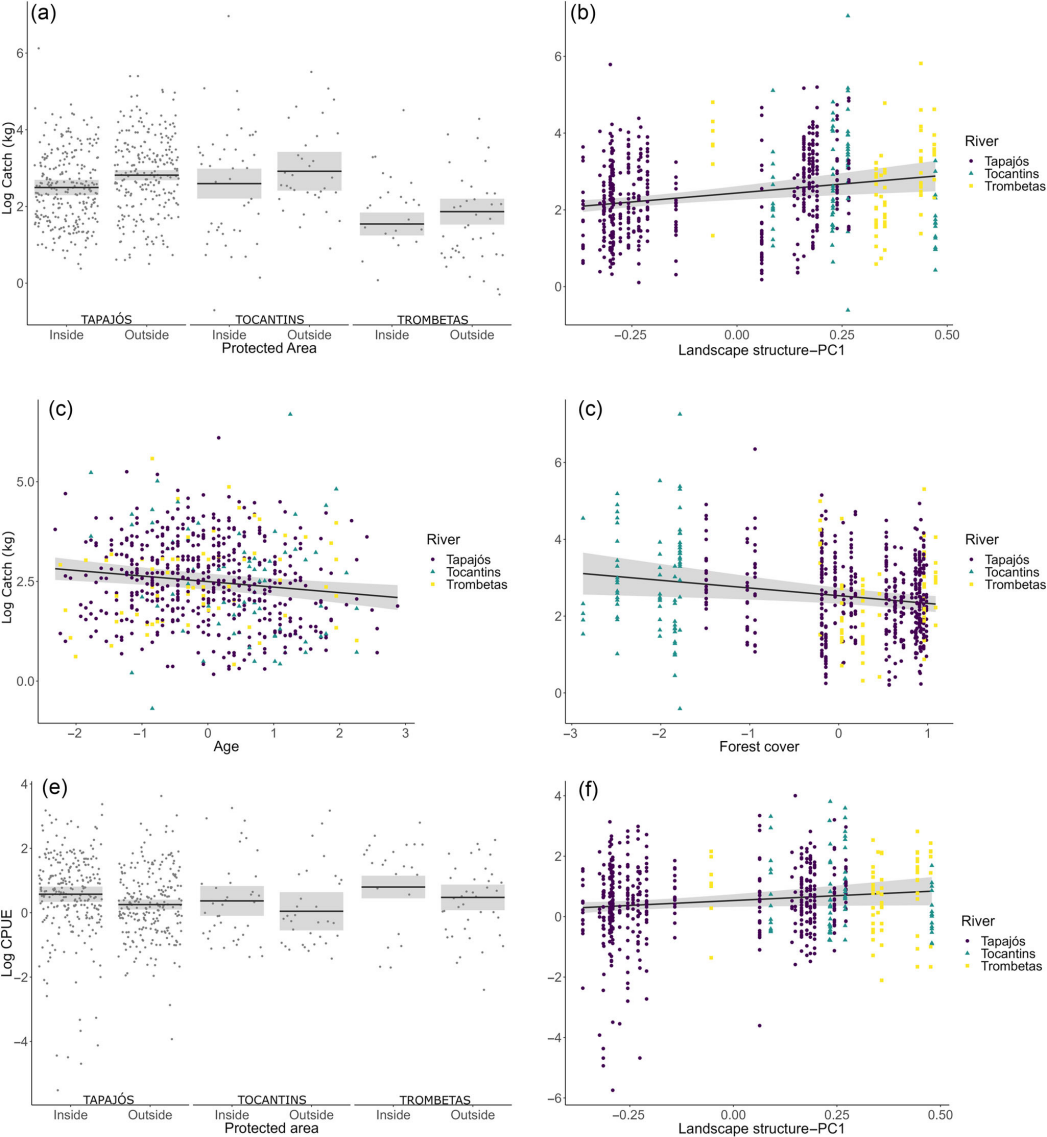
Significant relationships between predictor variables and response variables (fish catches and catch per unit effort [CPUE]) based on fisher interviews: (a) catches inside and outside protected areas (PAs) in the 3 rivers; (b) relationship between catches and landscape structure—PC1 (combination of landscape shape index, water area, and river width); (c) relationship between catch and fisher age; (d) relationship between catch and forest cover; (e) CPUE inside and outside PAs in the studied rivers; and (f) relationship between CPUE and the landscape structure—PC1 (points, an interview; black lines, mean in [a] and [e] and regression slopes in [b], [c], [d], and [f]; shaded areas, standard deviations in [a] and [e] and confidence intervals in [b], [c], [d], and [f]).

Model averaging of fish indicators based on interviews with fishers highlighted that predictors consistently showed positive or negative influences on each response variable (Table 3; Appendix S8). The predictors river, landscape structure (PC1), interviewee's age, PA, and forest cover influenced fish catches (Figure 3a). We observed lower catches in the Trombetas River (effect size −0.96) and higher catches outside PAs (0.33) (Figure 4a). We also detected positive relationships between catches and landscape structure (PC1) (0.96) (Figure 4b) and negative relationships with fishers’ age (−0.13) (Figure 4c) and forest cover (−0.2) (Figure 4d). The CPUE (Figure 3b) was lower outside PAs (−0.27) (Figure 4e) than inside and was positively associated with landscape structure (PC1) (0.66) (Figure 4f).

**TABLE 3:** Relative importance of 5 predictor variables explaining the variation in fisheries indicators (response variables) based on fisher knowledge.

Predictors	Fish catch (kg)	CPUE^a^
River	1.00^b^	0.37
Landscape structure—PC1^c^	0.99^b^	0.82^b^
Age	0.99^b^	0.30
Forest cover	0.93^b^	0.29
Protected area	0.93^b^	0.76^b^

^a^ Catch per unit effort, measured as kilograms of fish per hours of fishing × number of fishers.

^b^ Predictor variable showing consistent influence on the response variable.

^c^ Principal component 1, combination of landscape shape index, water area, and river width in a multivariate analysis.

## DISCUSSION

Our results underscore the relevance of SSFs in the studied rivers, where the average daily fish harvest per fisher was 28.3 kg based on interviews and 10.7 kg based on fish landings. Data from fish landings in the Tapajos River indicated that nearly one third of the harvest was consumed by fishers and their families, contributing directly to food and nutritional security in the region. In many low‐ and middle‐income countries, interviews often provide the only available data on the socioeconomic relevance of freshwater SSFs (Fluet‐Chouinard et al., 2018). These fisheries offer high‐quality and affordable food because freshwater fish supply proteins and essential micronutrients that improve the nutrition of vulnerable and impoverished populations in the Amazon (Heilpern et al., 2021) and other tropical regions (Béné et al., 2015). Conversely, seasonal shortages in fish harvests can exacerbate food insecurity in the Amazon (Tregidgo et al., 2020). In the Juruá River, a white‐water tributary of the Amazon River, households alternate between fishing during the low‐water season and hunting during the high‐water season (Begossi et al., 1999; Endo et al., 2016). In this river, fishing is more reliable and hunting compensates for the decline in fish catches when waters rise (Endo et al., 2016). Future researchers could explore whether the reliance on fish we observed is similarly associated with hunting intensity in clear‐water rivers.

The composition of fish harvests in tropical multispecies fisheries, such as those in the Amazon Basin, can be influenced by regional fish diversity (Heilpern et al., 2022), market‐driven selectivity (Hallwass & Silvano, 2016; Maccord et al., 2007), and cultural preferences (Begossi et al., 2004). In our study, a core group of fish species (Table 2), including tucunaré, pescada, jaraqui, aracu, and pacu, were among those most frequently cited by fishers across the 3 rivers and the most commonly recorded in fish landings from the Tapajos. These fish species, which were readily remembered and cited, can be considered culturally important (Freitas et al., 2020) in the studied rivers. Identification of culturally significant fish species can inform management strategies and impact assessments and thus guarantee long‐term food security in these riverine systems.

From a management perspective, fishers could target the more abundant fish species in each river, although this may be mediated by market preferences or cultural norms. A previous study, in which fish sampling and fisher interview data were compared, showed that, although pescada is abundant in the Tapajos and Tocantins Rivers, it is more frequently cited by fishers in the Tapajos (Dutra et al., 2023). Another promising management practice, adopted by the community of Pini (number 33 in Figure 1) in the Tapajos River, involves protecting lakes and managing populations of valuable sedentary species, such as tucunaré, which is one of the most cited and harvested fish in all 3 clear‐water rivers. Comanagement strategies based on lake closures and regulation of fishing effort have proven effective in increasing fish abundance, average size, and fishing yields in the Tocantins River (Silvano et al., 2014).

Aquatic contamination poses a growing concern for the consumption of some fish species in the studied rivers. Illegal gold mining and deforestation have raised mercury levels in Tapajos River; fish and humans show high levels of bioaccumulation of mercury (de Vasconcellos et al., 2021; Faial et al., 2015; Lino et al., 2019). The high levels of mercury could restrict the consumption and sale of large piscivorous fish species important to fisheries, such as filhote, dourada, and tucunaré (Appendix S4) (Pereyra et al., 2024). Although large‐scale industrial mining also occurs in the Trombetas River, its effects on fisheries are poorly studied. A recent proposal to reconcile food security, conservation, and public health in the Amazon recommends redirecting fisheries to smaller, lower‐trophic‐level fish species, such as the detritivorous jaraqui and branquinha (*Psectrogaster* spp.), which offer high nutritional content, contain low levels of contamination, and are less vulnerable to overfishing (Heilpern et al., 2025). This recommendation partially aligns with the culturally important species we identified in the clear‐water rivers. For example, jaraqui is a culturally significant species in the Tapajos and Tocantins Rivers, although branquinha is not, and neither species ranks among the most cited in the Trombetas River. Nevertheless, other low‐trophic‐level characins, such as pacus and aracus, are among the most cited in the studied rivers and may have lower contamination risk (Pereyra et al., 2024), offering management options aligned with cultural preferences and public health.

A substantial share of the fish species used in the 3 studied rivers (16 out of the 21 most cited) consisted of migratory fishes, including 11 species of longitudinal migrators and 5 of lateral migrators (Appendix S4). These migratory fishes provide multiple ecosystem services, such as seed dispersal, nutrient cycling, and food provision (Pelicice et al., 2023), but are also susceptible to impacts from overfishing and habitat alteration (Duponchelle et al., 2021). Our findings offer new insights into the proportion of migratory fishes harvested in the Tapajos and Trombetas Rivers, highlighting the importance of these migratory species in clear‐water rivers, as previously documented in other parts of the Amazon (Duponchelle et al., 2021).

The typically lower productivity of clear‐water rivers relative to white‐water rivers may limit the capacity of clear waters to sustain stocks of large fish species, except for migratory catfishes harvested in the main river channels (Hallwass, Schiavetti, et al., 2020; Hallwass, Silva, et al., 2020). Our harvest records and interview data corroborate previous information supplied by fishers, indicating migrations of large catfishes, such as filhote, across the Tapajos River (Nunes et al., 2019), and illustrating the complex migratory behavior of these species (Hegg et al., 2015). In other regions of the Amazon, overfishing has affected populations of these large migratory catfishes (Petrere et al., 2004; Prestes et al., 2022). The absence of clear territorial boundaries makes the management of longitudinal and long distance migratory fishes particularly challenging, often requiring basin‐wide management approaches (Duponchelle et al., 2021; Goulding et al., 2019; Hallwass & Silvano, 2016). These efforts so far have yet to be successfully implemented at the necessary scale. In response to demands from local communities, a broadscale fishing agreement has recently been established in the Tapajos River, encompassing the 10‐km areas of the 2 studied PAs (Figure 1). This agreement aims to regulate the fishing intensity of commercial boats (unpublished Portaria N° 2816/2022) and incorporates participatory fisheries monitoring and, given its extensive spatial coverage, offers the potential to contribute to the protection of migratory fishes. Our results provide a baseline and a justification for promoting such initiatives in oligotrophic clear‐water rivers, which represent about one third of the Amazonian basin (Goulding et al., 2003) and are where fisheries predominantly rely on migratory fish species (this study).

The results of our collaborative research are important for understanding how SSFs respond to, or may be affected by, ongoing and potential environmental changes in the studied rivers. Among these, the construction of existing and planned dams represents one of the main threats to fisheries based on migratory species in these clear‐water rivers. Although the Tapajos River remains free of dams, its fish populations and fisheries are vulnerable to planned hydropower projects and dams, especially if decision makers overlook downstream effects (Latrubesse et al., 2017; Runde et al., 2020). For example, a large dam was proposed near the studied community of São Luiz do Tapajos (Fearnside, 2015; Runde et al., 2020), where fisheries primarily targeted longitudinal migrators. These migratory fish species, highly valued by fishers, are also those most affected by river fragmentation because dams can block migratory routes essential for completing their life cycles (Duponchelle et al., 2021; Hallwass et al., 2013; Hoeinghaus et al., 2009).

As seen in the Brazilian Amazon and elsewhere, dams can also alter downstream hydrological regimes (Baird et al., 2021; Winemiller et al., 2016) and thus have cascading effects on floodplain connectivity and fish access to critical lateral habitats for feeding or reproduction. This disruption of fish lateral migrations and access to floodplains to feed on fruits has been documented in regions downstream from the Belo Monte dam on the Xingu River (Quaresma et al., 2025). The Trombetas River rapids have likewise attracted interest for hydropower development, although information on its fisheries and fish populations remains scarce (Ferreira, 1993; Isaac et al., 2015), which limits the capacity to assess the full extent of potential impacts. The Tocantins River, already subject to extensive deforestation and damming (Figure 1), stands among the most heavily altered and threatened rivers in the Brazilian Amazon (Pelicice et al., 2021; Swanson & Bohlman, 2021). Our findings indicated that fishers in the Tocantins still commonly harvest migratory fishes. However, future infrastructure projects, including new dams and a planned navigable waterway (Akama, 2017), threaten to further disrupt fish migrations and undermine the sustainability of SSFs in this river.

Our data can contribute to more comprehensive impact assessments that properly consider the needs and livelihoods of local fishers (Castro‐Diaz et al., 2018; Doria et al., 2017). For example, much of the scientific research on, and monitoring of, the effects of dams has been conducted after large dams were built as part of compensation measures (Baird et al., 2021). This was the case with the Belo Monte Dam (Pezzuti et al., 2024), which had a severe negative effect on SSFs and food security along the Xingu River (Lopes et al., 2024). Our results therefore reinforce the urgency of adopting a precautionary, participatory research approach in which fishers are collaborated with to generate critical baseline data before irreversible environmental changes occur. Despite basin‐wide planning efforts for hydropower expansion in the Amazon based on multiple indicators (Flecker et al., 2022), our results highlight the importance of considering the socioeconomic relevance of regionally distinctive fisheries, particularly in clear‐water rivers.

The interviewed fishers reported higher fish catches outside the PAs across the 3 rivers, which can be attributed to more intense fishing effort (Appendix S9), as previously observed in the Tapajos River (Hallwass, Silva, et al., 2020; Keppeler et al., 2020). This trend may reflect the occurrence of more commercially oriented fisheries outside PAs, in contrast to more diversified income opportunities in PAs. Many fishers interviewed in the middle Tapajos, an area without PAs, sell their catches outside their communities (Runde et al., 2020). Conversely, fishers reported higher CPUE inside PAs, suggesting either greater fish availability or improved conditions for harvesting fish in these PAs. In addition to influencing catches and CPUE, the PAs in the Tapajos River also affected the composition of fish used by interviewed fishers. These effects from PAs likely resulted from a combination of factors, including rules that restrict fish commercialization or certain kinds of fishing practices (e.g., gillnets and spearfishing), differences in local fish species availability, and cultural or household preferences. The specific regulations governing fisheries differ among the studied PAs: 3 restrict fishing to local residents, 2 limit fishing to domestic consumption, and 6 prohibit fishing techniques considered more harmful to fish stocks (Table 1). Although we lacked information on rule compliance, restrictions on commercial fishing and on fishing by outsiders may help reduce overall fishing effort in some of these PAs. These findings align with broader evidence on the benefits of PAs and comanagement initiatives involving local communities in the Amazon (Campos‐Silva et al., 2021; Silvano et al., 2014) and in SSFs globally (Koning et al., 2020; Kura et al., 2023; Muallil et al., 2019).

Forest cover has been positively associated with fish abundance and catches in the extensive floodplains of the more productive white‐water Amazon River (Arantes et al., 2018; Castello et al., 2018). However, we did not observe this relationship in the studied clear‐water rivers, where, according to interviews’ data, fish catches were inversely related to forest cover. This pattern may be explained by the limited floodplains in these rivers (compared with white‐water rivers) and the predominance of fisheries targeting migratory fish species, which may spend less time associated with flooded forests. Additionally, the widespread use of gillnets in the Tapajos River (Hallwass et al., 2023) may reduce fishing efficiency in densely vegetated flooded forests. Despite this, forest cover remained relevant to fisheries. In the Trombetas River, for example, the extensive forested areas associated with PAs (Figure 1) likely supported harvests of some species, such as pacu, that undertake lateral migrations between the river channel and flooded forests (Fernandes, 1997). Moreover, forests may be important in the long term; trophic models project that continued deforestation could reduce the abundance of commercial fish species in the Tapajos River over the next 50 years (Capitani et al., 2021). This reinforces the need for integrated forest and fisheries management in clear‐water rivers.

We provided firsthand evidence of a positive influence of river landscape complexity and heterogeneity on fish catches and CPUE, likely linked to greater habitat diversity in river stretches with islands and rock outcrops (Appendix S10). This finding has implications for impact assessment in the studied rivers. For example, landscape heterogeneity can be reduced by the demolition of rock outcrops in the Tocantins River (Akama, 2017) and by potential hydrological alterations downstream from proposed dams in the Tapajos River. We also observed lower average catches in the more pristine Trombetas River. In the absence of long‐term fisheries data to assess fish stock status, we suggest that these lower catches may reflect less commercially oriented fisheries because local people have alternative income sources, including recreational fishing and royalties from the mining industry. These factors warrant further investigation.

Older fishers reported lower catches than younger fishers, contrary to trends usually observed in coastal fisheries, where older fishers often perceive higher catches than younger fishers (Sáenz–Arroyo et al., 2005). This may be because some older fishers in our study may have reduced their fishing activity by the time of the interview or continued using less efficient techniques, such as paddled boats and hand lines (Hallwass, Schiavetti, et al., 2020).

Our results highlight the potential of collaborating with local fishers through interviews and voluntary monitoring. Although our study has limitations, such as providing a snapshot of fisheries mostly from the Tapajos River and not capturing seasonal variations, fishers can reliably recall an average fishing day (Castello et al., 2024), despite daily, seasonal, and interannual fluctuations. Limitations of the participatory monitoring included turnover among participating fishers (some left and others joined during sampling) and the need to double‐check data and exclude some records. Advantages included capacity building, greater fisher involvement in the research, and a larger dataset than typically obtained through surveys by field scientists (Silvano & Hallwass, 2020).

Other studies have further involved local people through in‐depth interviews, focus groups, and cocreated research (Esmail et al., 2023; Mekuria et al., 2024; Oloriz & Parlee, 2020). For example, Indigenous and riverine people documented environmental changes in Xingu River floodplains after dam construction and partnered with scientists to publish the findings (Quaresma et al., 2025). Although valuable locally, such inclusive research may be difficult to replicate at broader scales. We provided a broadscale fisheries assessment and comparison among rivers through rapid, systematic interviews combined with a participatory monitoring protocol applied across multiple riverine communities. Such data offer a critical baseline for informing management strategies that can be promptly implemented (Danielsen et al., 2021) and can be used to improve impact assessments (Runde et al., 2020). Compared with more localized in‐depth surveys, the rapid and systematic approach we used enabled broader research coverage, increasing its potential influence on decision‐making. This initial collaboration could set the stage for more inclusive fisheries management and conservation policies rooted in the knowledge and participation of local fishers.

## Supporting information



Supporting Information
