# Supplementary material for: Importance and spatial patterns of invisible fisheries in Amazonian clear‐water rivers as revealed by fisher knowledge and collaboration
Source: Conserv Biol. 2025 Oct 26;40(1):e70164. doi: 10.1111/cobi.70164 (PMC12856822; doi:10.1111/cobi.70164)
Supplement: Supplementary file 1 — Supporting Information [file COBI-40-e70164-s001.docx]

**Supporting information**

**Appendix S1.** Number of communities sampled per year of study. Some communities were sampled in multiple years. The amount of data collected (fish landings and interviews) per community is shown in Table 1; community locations are in Fig. 1.

| River | 2013 ^a^ | 2016 | 2018 | 2019 | 2022 | 2023 | Revisited ^c^ | Total ^d^ |
| --- | --- | --- | --- | --- | --- | --- | --- | --- |
| Tapajos | 9 ^b^ | 9 ^a,b^ | 12 ^b^ | 0 | 8 ^a,b^ | 17 ^b^ | 25 | 28 ^e^ |
| Tocantins | 0 | 0 | 4 ^b^ | 0 | 0 | 5 ^b^ | 4 | 5 |
| Trombetas | 0 | 0 | 0 | 4 ^b^ | 0 | 4 ^b^ | 2 | 6 |
| Total | 9 | 9 | 16 | 4 | 8 | 26 | 31 | 39 |

**^a^** Participatory monitoring of fish landings.

^b^ Interviews.

^c^ Number of communities that we revisited and sampled in more than one year. In such cases, we added new interviews made in more recent years to the existing data base. In some communities, we conducted participatory monito in one year, and interviews in another year.

^d^ Total number of distinct communities studied in each river, after controlling for revisited communities.

^e^ We revisited two communities in the Tapajos in three distinct years.

**Appendix S2.** Typical questionnaire used in interviews with fishers, translated into English (see Supplementary Material in Runde et al., 2020). Interviews were conducted in the interviewee’s native language (Brazilian Portuguese). Italicized text provides guidance for the interviewer. Questions analyzed in this study (1, 2, 3, 4, 5, 23, 24, 25) are in **bold.**

| INTERVIEW IN RIVER: _________________ | | | | | | | | | | | | | | | | | | | | | | | | | | | | | | | | Date:___/___/___ | | | | |
| --- | --- | --- | --- | --- | --- | --- | --- | --- | --- | --- | --- | --- | --- | --- | --- | --- | --- | --- | --- | --- | --- | --- | --- | --- | --- | --- | --- | --- | --- | --- | --- | --- | --- | --- | --- | --- |
| Socio-economic profile | | | | | | | | | | | | | | | | | | | | | | | | | | | | | | | | | | | | |
| **1) Community:** | | | | | | | | | **2) Interviewee:** | | | | | | | | | | | | | | | | | | | | | | | | | | **3) Age:** | |
| **4) Birth place/Where are you from:** | | | | | **5) Years lived in the community: ___ years** | | | | | | | | | | | | | | | | | | | | | | | 6) Are you the head of the household? ( ) Yes; ( ) No 🡪Who is? | | | | | | | | |
| 7) How many people, including you, live in your house? | | | | | | | | | | | | | | | | | | | | | | | | | | | | | | | | | | | | |
| 8) Education (of family members) (*circle education of the head of household*🡪) | | | | | | | | | None | | | | | | | | Primary | | | | | | | | | Secondary | | | | | Higher | | | | | |
| Age/Sex | | | | | | | | | ♂ | | | ♀ | | | | | ♂ | | | | ♀ | | | | | ♂ | | | | ♀ | ♂ | | | | | ♀ |
| ( ) ≤5 | | | | | | | | |  | | |  | | | | |  | | | |  | | | | |  | | | |  |  | | | | |  |
| ( ) 6-14 | | | | | | | | |  | | |  | | | | |  | | | |  | | | | |  | | | |  |  | | | | |  |
| ( ) 15-30 | | | | | | | | |  | | |  | | | | |  | | | |  | | | | |  | | | |  |  | | | | |  |
| ( ) 31-64 | | | | | | | | |  | | |  | | | | |  | | | |  | | | | |  | | | |  |  | | | | |  |
| ( ) ≥65 | | | | | | | | |  | | |  | | | | |  | | | |  | | | | |  | | | |  |  | | | | |  |
| 9) Does your house have/has (a): ( ) Bathroom; ( ) TV; ( ) Fridge/Freezer; ( ) Running water; ( ) Electric light(s); ( ) Internet; ( ) Computer | | | | | | | | | | | | | | | | | | | | | | | | | | | | | | | | | | | | |
| 10) Are you participating in an organisation or cooperation (of any sort)? ( ) No; ( ) Yes 🡪 Which: | | | | | | | | | | | | | | | | 11) Are you receiving any governmental benefits? ( ) No; ( ) Yes  🡪Which? | | | | | | | | | | | | | | | | | | | | |
| 12) What is the average monthly income? (*if possible,* *indicate value in R$*): ( )0-500; ( )501-1000; ( )1001-1500; ( )1501-2000; ( ) > 2000 | | | | | | | | | | | | | | | | | | | | | | | | | | | | | | | | | | | | |
| Use of resources & economic activities | | | | | | | | | | | | | | | | | | | | | | | | | | | | | | | | | | | | |
| 13) Which economic activities do you pursue? | | | | | | Order of importance 1 (highest), 2,3... | | | | | | | | | | | | | | | | | | | | | 14) Does anyone else in the family contribute to the household income? What is this person/what are they doing? | | | | | | | | | |
|  |  |  |  |  |  | Dry season | | | | | | | | | Wet season | | | | | | | | | | | |  |  |  |  |  |  |  |  |  |  |
| ( ) Fishing | | | | | |  | | | | | | | | |  | | | | | | | | | | | |  | | | | | | | | | |
| ( ) Hunting | | | | | |  | | | | | | | | |  | | | | | | | | | | | |  |  |  |  |  |  |  |  |  |  |
| ( ) Livestock farming | | | | | |  | | | | | | | | |  | | | | | | | | | | | |  |  |  |  |  |  |  |  |  |  |
| ( ) Non-timber forest products: *which?* | | | | | |  | | | | | | | | |  | | | | | | | | | | | |  |  |  |  |  |  |  |  |  |  |
| ( ) Others: | | | | | |  | | | | | | | | |  | | | | | | | | | | | |  |  |  |  |  |  |  |  |  |  |
| ( ) Others: | | | | | |  | | | | | | | | |  | | | | | | | | | | | |  |  |  |  |  |  |  |  |  |  |
|  | | 15) Importance for diet (*number*) | | | | | | | | | | | | | | | | | | | | | | | | | | | | | | | | | | |
|  | | Fish | | | | | | | Game meat *(which animal do you hunt?)* | | | | | | | | | Chicken | | | | | Beef | | | | | | River turtle | | | | | Pig | | |
| Dry season | |  | | | | | | |  | | | | | | | | |  | | | | |  | | | | | |  | | | | |  | | |
| Wet season | |  | | | | | | |  | | | | | | | | |  | | | | |  | | | | | |  | | | | |  | | |
| Fishing | | | | | | | | | | | | | | | | | | | | | | | | | | | | | | | | | | | | |
| 16) When did you start fishing on your own? (*insert age*): | | | | | | | | | | | | | | | | | | | 27) How many days do you fish per week: | | | | | | | | | | | | | | | | | |
| 18) Type & number of fishing vessel(s) | | | | Size | | | Does it have a motor? | | | | Dif. to when you started fishing? How? | | | | | | | | 19) Which fishing equipment do you use *and how many do you have *(insert number (1)(2)(...))* | | | | | | | | | | | | | | Dif. to when you started fishing? How? | | | |
| ( ) Canoe | | | |  | | |  | | | |  | | | | | | | | ( ) Fishing net (gillnet)  - Mesh size: | | | | | | | | | | | | | |  | | | |
| ( ) Bajara | | | |  | | |  | | | |  | | | | | | | | ( ) Cast net: | | | | | | | | | | | | | |  | | | |
| ( ) Boat with integrated ice-storage capacity | | | |  | | |  | | | |  | | | | | | | | ( ) Hook & Line | | | | | | | | | | | | | |  | | | |
| ( ) Boat without integrated ice-storage capacity | | | |  | | |  | | | |  | | | | | | | | ( ) Rod | | | | | | | | | | | | | |  | | | |
| ( ) Others: | | | |  | | |  | | | |  | | | | | | | | ( ) Longline | | | | | | | | | | | | | |  | | | |
|  | | | |  | | |  | | | |  | | | | | | | | ( ) Harpoon | | | | | | | | | | | | | |  | | | |
|  | | | |  | | |  | | | |  | | | | | | | | ( ) Trident | | | | | | | | | | | | | |  | | | |
|  | | | |  | | |  | | | |  | | | | | | | | ( ) Others: | | | | | | | | | | | | | |  | | | |
| 20) Fishing site | Lake | | | | | | | River channel | | | | | | Restinga | | | | | | | | River | | | | | | Forest stream | | | | | Others: | | | |
| Wet season |  | | | | | | |  | | | | | |  | | | | | | | |  | | | | | |  | | | | |  | | | |
| Dry season |  | | | | | | |  | | | | | |  | | | | | | | |  | | | | | |  | | | | |  | | | |
| 21) Where do you sell the fish? ( )  Within the community; ( ) Outside of the community (fishmonger, middleman); ( ) in the city; ( ) Others (*specify*): | | | | | | | | | | | | | | | | | | | | | | | | | | | | | | | | | | | | |
| 22) Since you started fishing: The value that you receive for selling the fish has: ( ) Increased; ( ) Decreased; ( ) Stayed the same | | | | | | | | | | | | | | | | | | | | | | | | | | | | | | | | | | | | |
| **23) How many kg of fish do you catch in a normal fishing day?**  **How many hours do you spend fishing? How many people participate in the fishing? *(write total number incl. fisher)*** | | | | | | | | | | | | | | | | | | | | | | | | | | | | | | | | | | | | |
| **24) How many kg of fish did you catch in a normal fishing day when you started fishing?**  **How many hours did you spend fishing? How many people participated in the fishing? *(write total number incl. fisher)*** | | | | | | | | | | | | | | | | | | | | | | | | | | | | | | | | | | | | |
| Current fish species | | | | | | | | | | | | | | | | | | | | | | | | | | | | | | | | | | | | |
| **25) 5 most important species (*note in the order that they speak)*** | | | **Order of importance** | | | | **Abundance since (you) started fishing** | | | | | | | | | | | | | | | | | **Why?** | | | | | | | | **Medium size (cm), (*show measuring tape to the interviewee)*** | | | | |
|  |  |  |  |  |  |  | **+** | | | | | | **-** | | | | | | | **=** | | | |  |  |  |  |  |  |  |  |  |  |  |  |  |
|  | | |  | | | |  | | | | | |  | | | | | | |  | | | |  | | | | | | | |  | | | | |
|  | | |  | | | |  | | | | | |  | | | | | | |  | | | |  | | | | | | | |  | | | | |
|  | | |  | | | |  | | | | | |  | | | | | | |  | | | |  | | | | | | | |  | | | | |
|  | | |  | | | |  | | | | | |  | | | | | | |  | | | |  | | | | | | | |  | | | | |
|  | | |  | | | |  | | | | | |  | | | | | | |  | | | |  | | | | | | | |  | | | | |
| Fish species when fishing was started | | | | | | | | | | | | | | | | | | | | | | | | | | | | | | | | | | | | |
| 26) 5 most important species (*note in the order that they speak*) | | | | | | | | | | Order of importance | | | | | | | | | | | | | | | Medium size (cm) *(show measuring tape*) | | | | | | | | | | | |
|  | | | | | | | | | |  | | | | | | | | | | | | | | |  | | | | | | | | | | | |
|  | | | | | | | | | |  | | | | | | | | | | | | | | |  | | | | | | | | | | | |
|  | | | | | | | | | |  | | | | | | | | | | | | | | |  | | | | | | | | | | | |
|  | | | | | | | | | |  | | | | | | | | | | | | | | |  | | | | | | | | | | | |
|  | | | | | | | | | |  | | | | | | | | | | | | | | |  | | | | | | | | | | | |
| 27) Have you seen any new fish since you started fishing? ( ) No; ( ) Yes Which? | | | | | | | | | | | | | | | | | | | | | | | | | | | | | | | | | | | | |
| Anthropogenic impacts & changes | | | | | | | | | | | | | | | | | | | | | | | | | | | | | | | | | | | | |
| 28) What do you think, which changes have happened or are happening in the region? When did the changes happen or when did they start? | | | | | | | | | | | | | | | | | | | 29) How do you or your community deal with the changes? | | | | | | | | | | | | | | | | | |
| 30) How do these changes affect your fishing? | | | | | | | | | | | | | | | | | | | 31) Do you think that the fishing for your children will be:  The same ( ); better ( ); worse ( ); Why? | | | | | | | | | | | | | | | | | |

Do you know other fishers who could participate in our study? Please provide names.

**Appendix S3.** Example of a participatory monitoring form for recording fish landings from a single fishing trip in the Tapajos River, translated into English (see Supplementary Material in Nagl et al., 2021). Original forms were in A4 format and written in Portuguese.


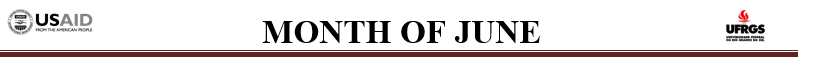


Fisherman’s name:_______________________________ Community:______________

**Date:** ___/___/___
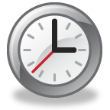
 **Start hour**:__________ **Finish hour**:____________

**FISHING SPOT (name): Time of travel to the fishing spot:___________**

( ) Tapajós River ( ) Lake:_______________ ( ) Igarapé:____________

( ) Other:_____________________________

**FISHING GEAR**

( ) gillnet: Mesh size:___________ number of gillnets:___________

( ) Line or pole and line ( ) Longline: number of hooks:__________

( ) Spear ( ) Castnet ( ) Others:_______________

**BOAT**

( ) Paddle boat ( ) Boat ( ) Longtail powered boat ( ) Large motor boat ( ) Other:_____

**CAPTURE** ****

| **Fish (local name)** | **Number of fish** | **Total weight (Kg)** | **Smallest Size (cm)** | **Largest Size (cm)** | **Number with eggs** |
| --- | --- | --- | --- | --- | --- |
|  |  |  |  |  |  |
|  |  |  |  |  |  |
|  |  |  |  |  |  |
|  |  |  |  |  |  |
|  |  |  |  |  |  |

**Sold**: ( ) Yes ( ) No 🡪 **Quantity:** _______**Kg**

**Amount received =** ___________ R$

**Consumed fish:** ( ) Yes ( ) No 🡪 **Quantity:** _______**Kg**

Other fishermen in the crew? ( ) Yes ( ) No 🡪 How many?________

How many Kg of fish captured by all?_______________ Kg.

**Appendix S4.** Fish species most cited or harvested by fishers in the three studied rivers (see Table 2), indicating their migratory behavior (Herrera‐et al., 2024) and potential mercury (Hg) contamination based on Pereyra et al. (2024).

| Fish common name | Fish species | Migratory behavior | Potential contamination by Hg |
| --- | --- | --- | --- |
| Acara | Cichlidae | Sedentary | No |
| Acaratinga | *Geophagus* spp. | Sedentary | No |
| Acari | Loricariidae | Sedentary | No |
| Aracu ^b^ | *Leporinus* spp., *Schizodon* spp. | Longitudinal | No |
| Charuto | *Hemiodus* spp. | Lateral | No |
| Corvina ^b^ | *Pachypops fourcroi* | Lateral | No |
| Curimata ^b^ | *Prochilodus nigricans* | Longitudinal | No |
| Dourada ^b,c^ | *Brachyplatystoma flavicans* | Longitudinal | Yes |
| Filhote ^b,c^ | *Brachyplatystoma filamentosum* | Longitudinal | Yes |
| Jaraqui ^b^ | *Semaprochilodus* spp. | Longitudinal | No |
| Jau | *Zungaro* sp. | Longitudinal | Yes |
| Mandi | *Pimelodus* spp. | Undetermined | No |
| Mapará ^b^ | *Hypophthalmus* spp. | Longitudinal | No |
| Matrinxã ^b^ | *Brycon* spp. | Longitudinal | No |
| Pacu ^b^ | *Myleus* spp., *Myloplus* spp. | Lateral | No |
| Pescada ^a,b,c^ | *Plagioscion squamosissimus* | Lateral | Yes |
| Piranha | *Pygocentrus* spp., *Serrasalmus* spp. | Lateral | Yes |
| Sarda ^b,c^ | *Pellona castelnaeana* | Longitudinal | Yes |
| Surubim | *Pseudoplatystoma* spp. | Longitudinal | Yes |
| Tambaqui ^b^ | *Colossoma macropomum* | Longitudinal | No |
| Tucunaré ^c^ | *Cichla* spp. | Sedentary | Yes |

**Appendix S5.** Detailed procedures for calculating predictor variables and for applying the model averaging approach.

1) Formula adopted to calculate the landscape shape index (LSI) for each studied community, following this sequence of analytical steps: first, define the study area; second, identify and calculate the perimeter and area of all islands and land masses within the study area; third, add up the perimeters and areas of all land masses; fourth divide the total perimeter by the total area to get the perimeter-to-area ratio; fifth multiply the perimeter-to-area ratio by 4π to get the LSI.


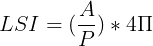


Where A is the total area of all land masses within the study area; P is total perimeter of all land masses within the study area.

2) Steps adopted in the model averaging approach (Burnham & Anderson, 2002). First, 32 multiple linear models with all possible combinations of fixed variables were fitted to the data. Second, we used corrected AICc to measure the plausibility of each candidate model. Third, we calculated the Akaike weight for each model and normalized across the set of candidate models, to sum to 1. Fourth, we used Akaike weights to obtain averaged estimates for each parameter. Fifth, we calculated the relative importance of each predictor variable by summing all Akaike weights over all models that include each predictor. We preferred a model averaging analysis instead of a classic model selection, due to the usually small differences between the best model and the other model candidates.

We used partial residual plots to visualize the significant effects, as these plots show the relationship between a given predictor variable and the response variable after considering the influence of other predictor variables in the model (Breheny & Burchett, 2017).

**Appendix S6.** Results (Global *R* and significance values) from pairwise tests in the two-way crossed analysis of similarities (ANOSIM) assessing differences in fish species composition among rivers and between fishing communities inside and outside protected areas (PAs) in the Tapajos River.

| Analysis | | Global *R* | *P* |
| --- | --- | --- | --- |
|  | Among rivers (global) | 0.872 | 0.001 |
| Pairwise tests | |  |  |
|  | Rivers |  |  |
|  | Tapajós and Tocantins | 0.892 | 0.001 |
|  | Tapajós and Trombetas | 0.888 | 0.001 |
|  | Tocantins and Trombetas | 0.602 | 0.04 |
|  | Inside and Outside PAs in the Tapajos | 0.316 | 0.001 |

a)


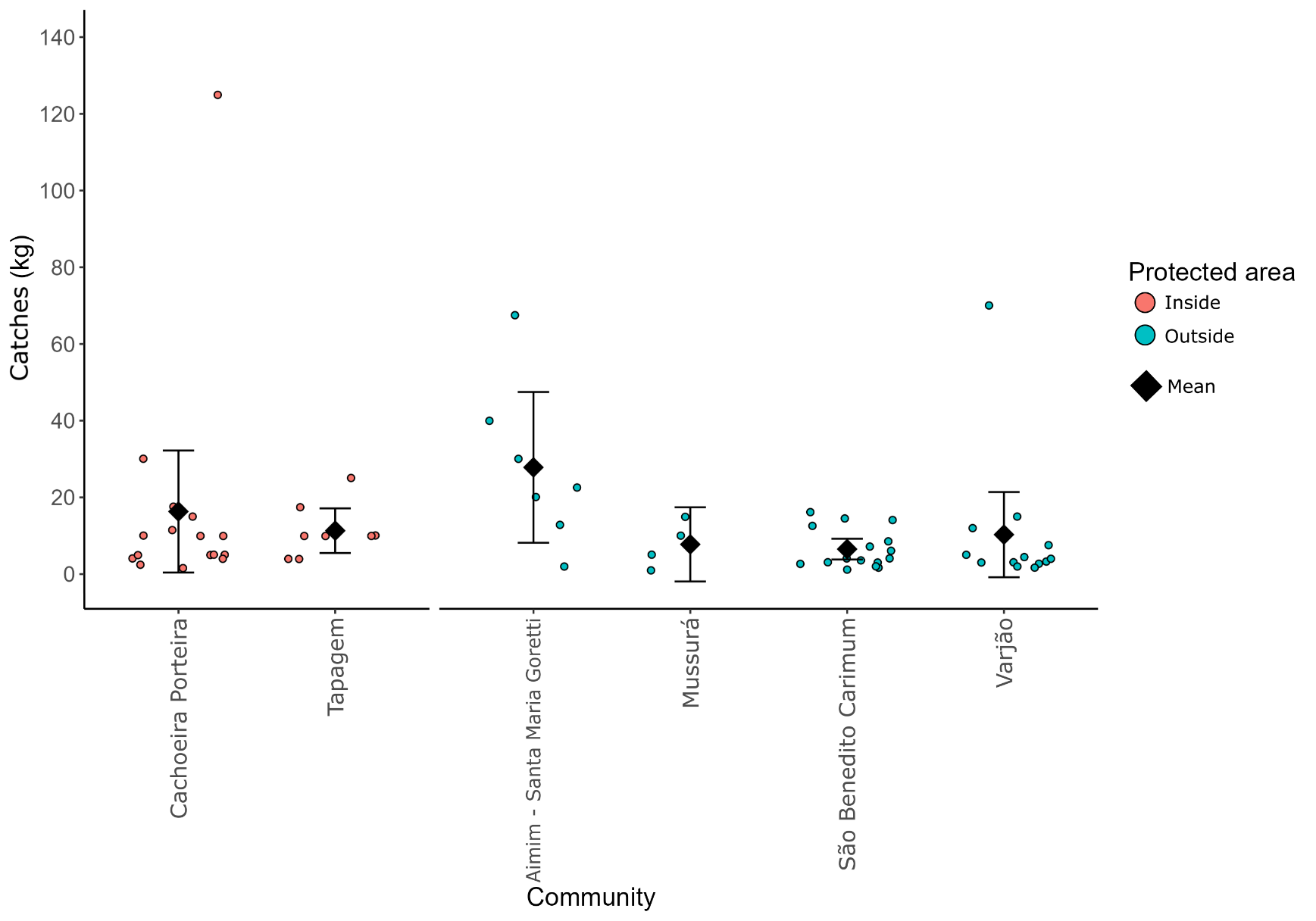


b)


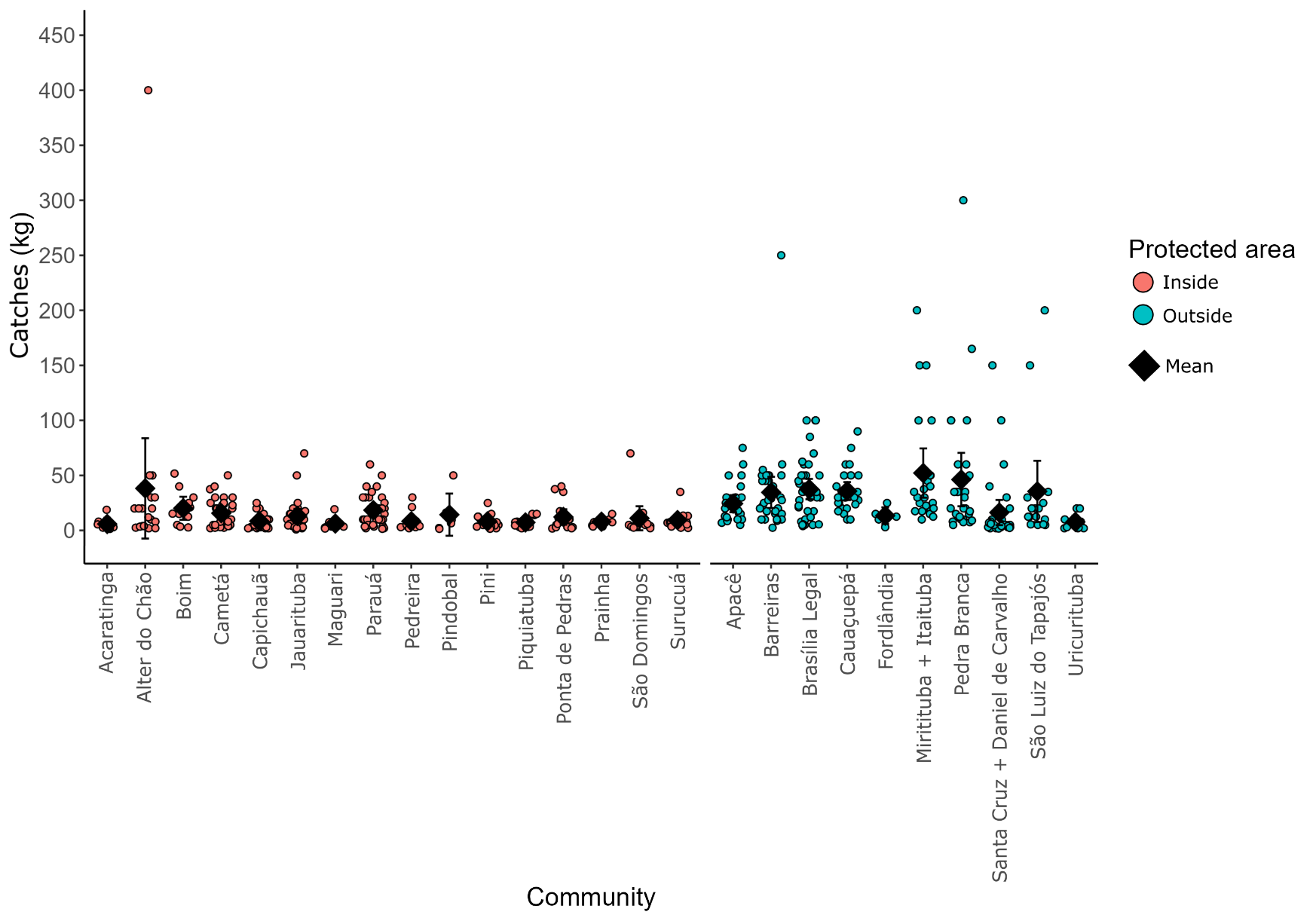


c)


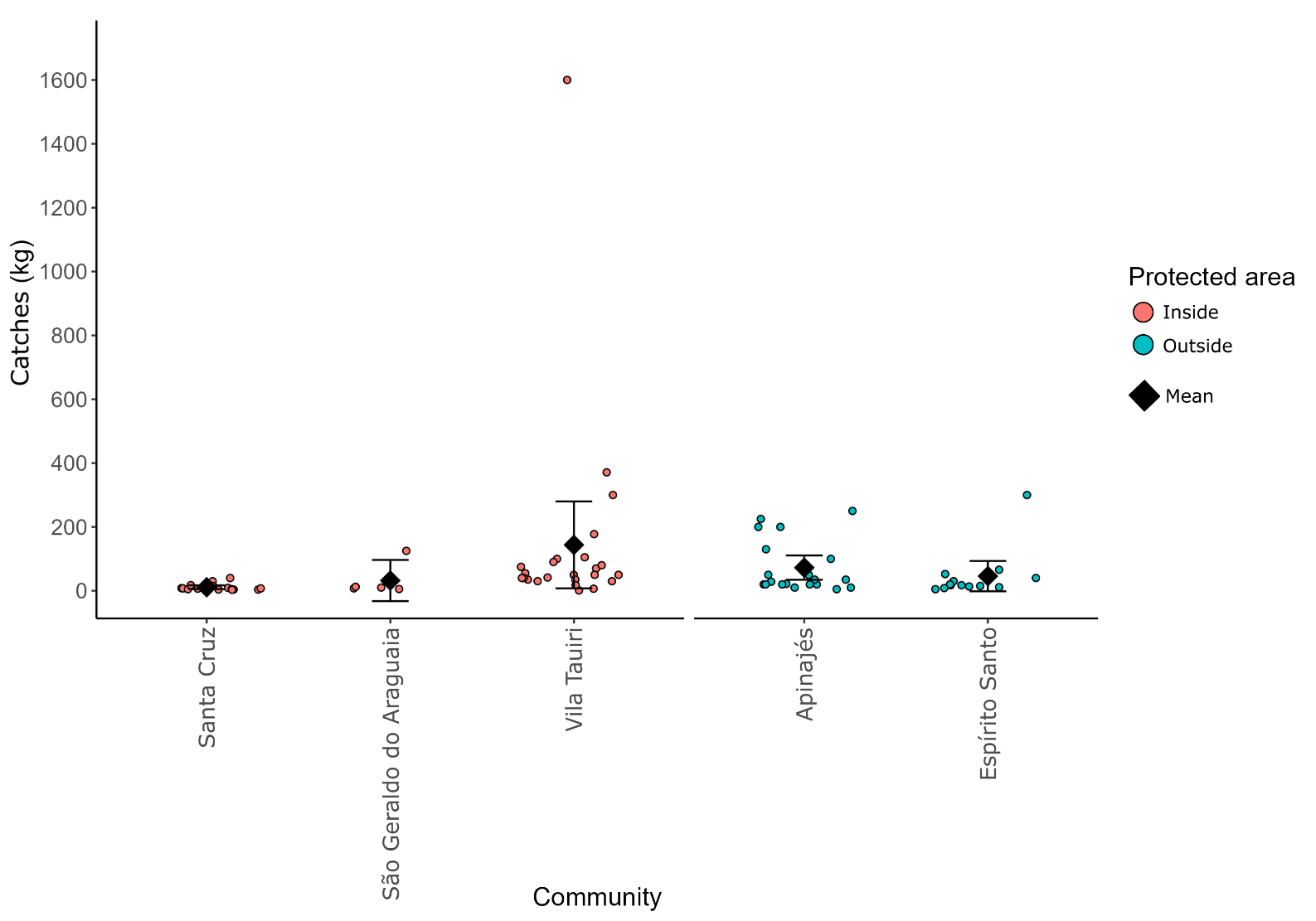


**Appendix S7.** Average fish catch (kg) reported by interviewed fishers in each studied community, inside and outside protected areas (PAs), across the three studied rivers: (a) Trombetas, (b) Tapajos, (c) Tocantins. Each dot represents a single interview.

**Appendix S8.** The four best models, ranked by AICc, for the response variables: fish catch (kg) and catch per unit of effort (CPUE). Landscape structure – PC1 represents the combination of three landscape attributes (landscape shape index, water area and river width) on the first axis (PC1) of a multivariate analysis. Age refers to the interviewee’s age.

| Models | AICc | Delta | Weight | R² adjusted |
| --- | --- | --- | --- | --- |
| *Fish catch (kg)* |  |  |  |  |
| Age + Forest cover + Landscape structure – PC1 + River + Protected area | 1888.0 | 0.00 | 0.834 | 0.19 |
| Age + Forest cover + Landscape structure – PC1 + River | 1892.8 | 4.80 | 0.075 | 0.18 |
| Age + Landscape structure – PC1 + River + Protected Area | 1893.0 | 5.00 | 0.068 | 0.18 |
| Forest cover + Landscape structure – PC1 + River + Protected area | 1896.7 | 8.62 | 0.011 | 0.18 |
| *CPUE* |  |  |  |  |
| Landscape structure – PC1 + Protected area | 2038.2 | 0.00 | 0.271 | 0.014 |
| Age + Landscape structure – PC1 + Protected area | 2039.8 | 1.63 | 0.120 | 0.013 |
| Forest cover + Landscape structure – PC1 + Protected area | 2040.2 | 2.03 | 0.098 | 0.013 |
| Landscape structure – PC1 + River + Protected area | 2040.8 | 2.63 | 0.072 | 0.013 |


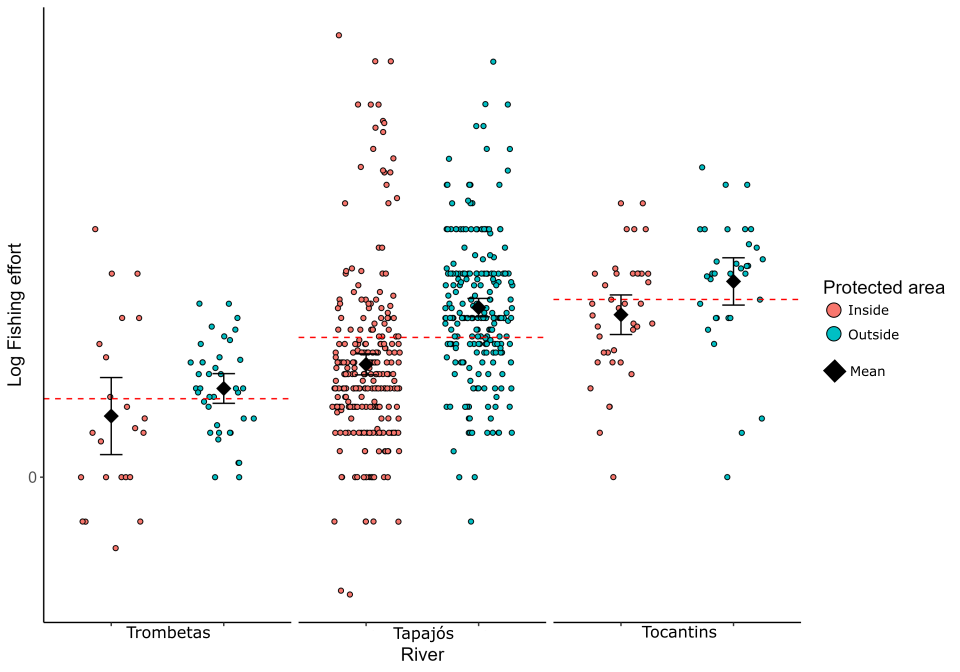


**Appendix S9.** Average fishing effort (hours fishing x number of fishers) based on interviews, inside and outside protected areas in the three studied rivers. The red line indicates the overall mean for each river.


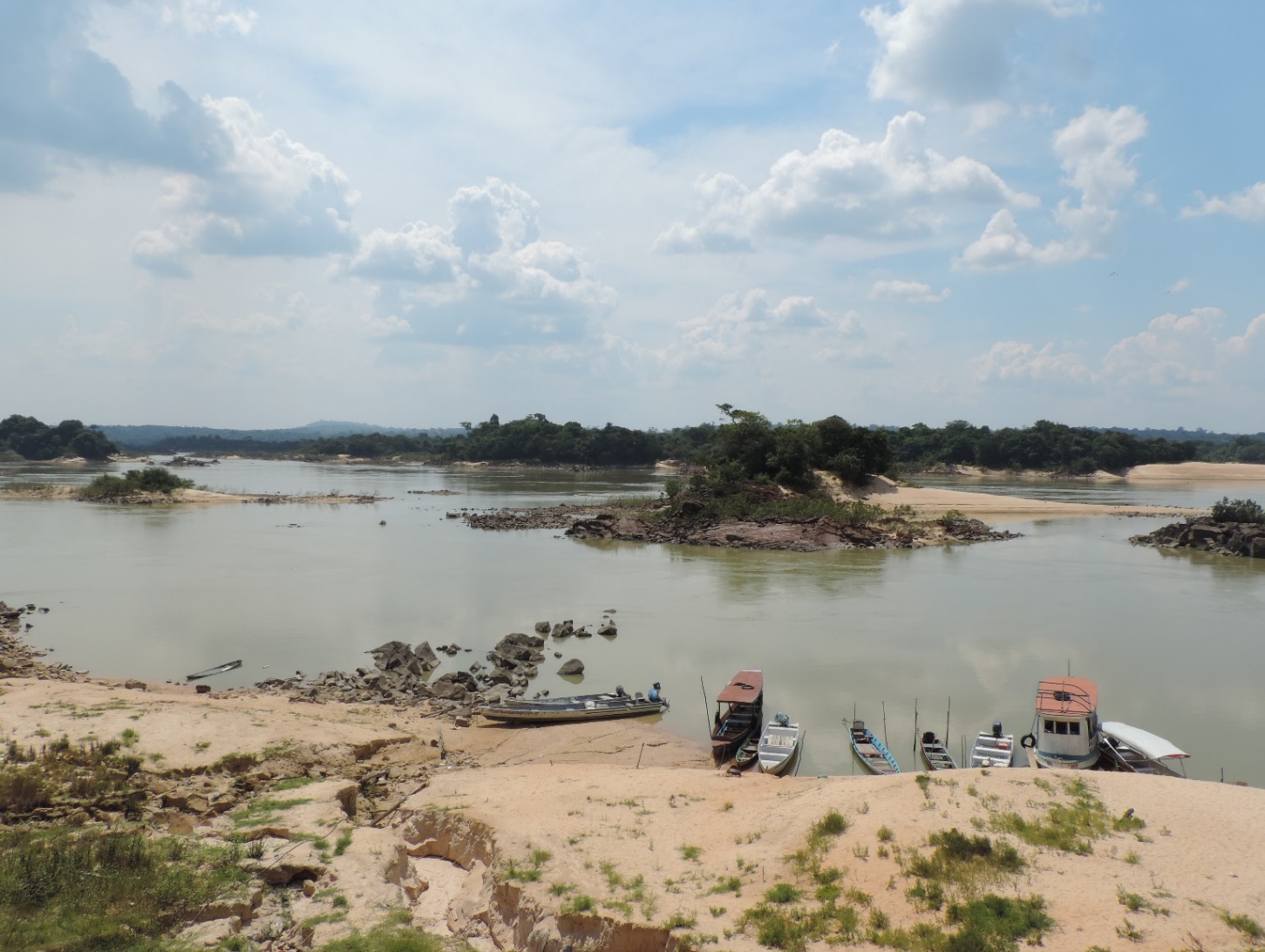


**Appendix S10.** Heterogeneous riverine landscape with islands, beaches, and rock outcrops during the dry season, in front of the fishing community of São Luiz do Tapajos, in the Tapajos River. This area can be affected by a planned dam upstream. Photo: Renato Silvano.

**References of Supporting information**

Breheny, P., & Burchett, W. (2017). Visualization of regression models using visreg. The R Journal, 9(2), 56-71.

Burnham, K. P., & Anderson, D. R. (2002). Model selection and multimodel inference. A Practical Information-Theoretic Approach, 2. Springer, New York

Herrera‐R, G. A., Heilpern, S. A., Couto, T. B., Victoria‐Lacy, L., Duponchelle, F., Correa, S. B., Farah‐Pérez, A., López‐Casas, S., Cañas‐Alva, C. M., & Doria, C. R. (2024). A synthesis of the diversity of freshwater fish migrations in the Amazon basin. Fish and Fisheries, 25(1), 114–133.

Nagl, P., Hallwass, G., Tomazoni‐Silva, L. H., Nitschke, P. P., Rowedder, A. R. P., Romero‐Martinez, A. T., & Silvano, R. A. M. (2021). Protected areas and frugivorous fish in tropical rivers: Small‐scale fisheries, conservation and ecosystem services. Aquatic Conservation: Marine and Freshwater Ecosystems, 31(10), 2752-2771.

Pereyra, P. E. R., Ospina‑Alvarez, A., Dutra, M. C. F., Schallenberger, B. H., Hallwass, G., Begossi, A., Clauzet, M., Lopes, P. F. M., & Silvano, R. A. M. (2024). Integrating fishers’ knowledge on fish use with ecological networks to assess the ‘silent threat’ of mercury contamination in tropical marine and freshwater fisheries. Environment, Development and Sustainability. https://doi.org/10.1007/s10668-024-04990-y

Runde, A., Hallwass, G., & Silvano, R. A. M. (2020). Fishers’ knowledge indicates extensive socioecological impacts downstream of proposed dams in a tropical river. One Earth, 2(3), 255–268.
